# Discriminating High from Low Energy Conformers of Druglike Molecules: An Assessment of Machine Learning Potentials and Quantum Chemical Methods

**DOI:** 10.1002/cphc.202400992

**Published:** 2025-02-27

**Authors:** Linghan Kong, Richard A. Bryce

**Affiliations:** ^1^ Division of Pharmacy and Optometry School of Health Sciences Manchester Academic Health Sciences Centre University of Manchester Oxford Road Manchester M13 9PT UK

**Keywords:** Ligand, high energy conformations, machine learning potentials, quantum chemistry

## Abstract

Accurate and efficient prediction of high energy ligand conformations is important in structure‐based drug discovery for the exclusion of unrealistic structures in docking‐based virtual screening and *de novo* design approaches. In this work, we constructed a database of 140 solution conformers from 20 druglike molecules of varying size and chemical complexity, with energetics evaluated at the DLPNO‐CCSD(T)/complete basis set (CBS) level. We then assessed a selection of machine learning potentials and semiempirical quantum mechanical models for their ability to predict conformational energetics. The GFN2‐xTB tight binding density functional method correlates with reference conformer energies, yielding a Kendall's τ of 0.63 and mean absolute error of 2.2 kcal/mol. As putative internal energy filters for screening, we find that the GFN2‐xTB, ANI‐2x and MACE‐OFF23(L) models perform well in identifying low energy conformer geometries, with sensitivities of 95 %, 89 % and 95 % respectively, but display a reduced ability to exclude high energy conformers, with respective specificities of 80 %, 61 % and 63 %. The GFN2‐xTB method therefore exhibited the best overall performance and appears currently the most suitable of the three methods to act as an internal energy filter for integration into drug discovery workflows. Enrichment of high energy conformers in the training of machine learning potentials could improve their performance as conformational filters.

## Graphical abstract

Machine learning and semiempirical quantum mechanical potentials are assessed for their ability to distinguish high from low energy conformations of druglike molecules. The GFN2‐xTB tight binding density functional method produces the best overall performance. Greater enrichment of high energy conformers in the training of machine learning potentials could improve their performance as conformational filters.

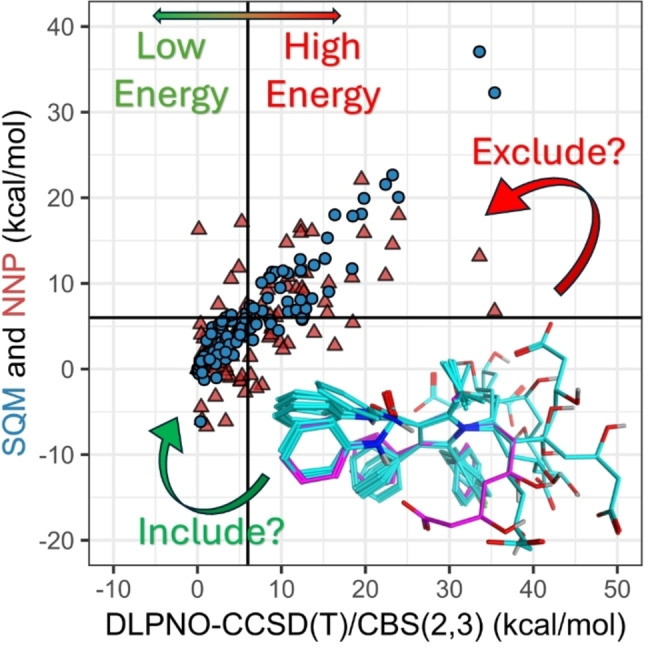

## 1 Introduction

An appreciation of the conformational energetics of small organic molecules is an essential element in the process of structure‐based drug design. In a study by Perola et al.,^[1]^ 60 % of a set of 150 ligands were estimated via molecular mechanics (MM) to bind to their target protein with an internal energy of under 5 kcal/mol, while 10 % exhibited strain energies exceeding 9 kcal/mol. Sitzmann et al.^[2]^ similarly employed a molecular mechanical method to estimate internal energies of protein‐bound ligand conformers, using the MMFF94s force field: this approach was applied to 415 small molecules, finding an average conformational energy of 6.7 kcal/mol with a relatively broad range of 0 to 25 kcal/mol. Potential sources of error in estimates of bound ligand strain include issues relating to experimental structure assignment such as resolution and the use of restraints.^[3]^ Alongside this, however, an inability of computational methods to accurately calculate the relative energetics of a ligand's conformers can lead to over‐ or underestimated protein binding affinities and thus increased virtual screening false negatives or positives respectively.^[4]^

A further challenge lies in the need for large scale conformer evaluation, thus requiring both accuracy and efficiency. Molecular mechanical force fields have consequently been a logical choice for estimating conformational energetics of druglike molecules in virtual screening and molecular docking,^[5]^ although its accuracy in this regard has proved to be limited.[^6^, ^7^] Alternatively, quantum mechanics (QM) can be employed to achieve more accurate conformational energies and geometries, but at considerably higher computational cost. High level QM models such as the coupled‐cluster singles, doubles and perturbative triples method, CCSD(T), cannot routinely provide large‐scale estimates of conformational energetics but can furnish useful benchmark datasets for assessing lower level methods.[^8^, ^9^, ^10^]

Density functional theory (DFT) methods scale more affordably^[11]^ with system size compared to CCSD(T). However, DFT is still considerably slower than semiempirical QM methods, based either on the NDDO approximation, such as the PMx models,^[12]^ or density functional tight binding approaches, such as the DFTB3 and the GFN2‐xTB approach.[^13^, ^14^, ^15^] Empirical correction schemes have been developed to improve the accuracy of these methods: popular schemes include the DFT‐D3 dispersion correction,^[16]^ H5 hydrogen bond correction^[17]^ and X halogen bond correction.^[18]^

The recent emergence of machine learning potentials (MLPs) has offered a competitive alternative to MM and QM methods.^[19]^ A widely used set of MLPs are the ANI suite, such as ANI‐2x and ANI‐1ccx,^[20]^ which are neural network potentials that employ atomic environment vectors (AEVs) to probe the chemical neighbourhood of atoms, and are trained on large numbers (10^5^–10^6^) of QM energies and geometries.^[21]^ The ANI methods have demonstrated their ability to make accurate relative energy predictions, with errors often within 1 kcal/mol to the reference DFT values, on small organic molecular systems.[^20^, ^21^, ^22^, ^23^, ^24^] In a study of monosaccharide pyranose ring puckers comparing the ANI‐1ccx, ANI‐2x and DFTB‐based GFN2‐xTB methods, ANI‐1ccx was found to provide correct relative energy predictions and accurate conformations in most cases, outperforming ANI‐2x and GFN2‐xTB.^[25]^ In another study of biaryl torsions of drug fragments, both ANI‐1ccx and ANI‐2x was found to reproduce the potential energy surfaces with mean errors ≤ 1 kcal/mol.^[26]^ Zengin et al.^[27]^ found that by employing ANI‐2x as a rescoring function for docking poses, it outperformed most conventional docking scoring functions. MLP methods such as ANI and the graph neural network (GNN)‐based MACE‐OFF approach,^[28]^ require computational resources similar to DFTB‐based methods,^[29]^ making them viable for evaluating conformational energies of small organic molecules.

**Figure 1:**
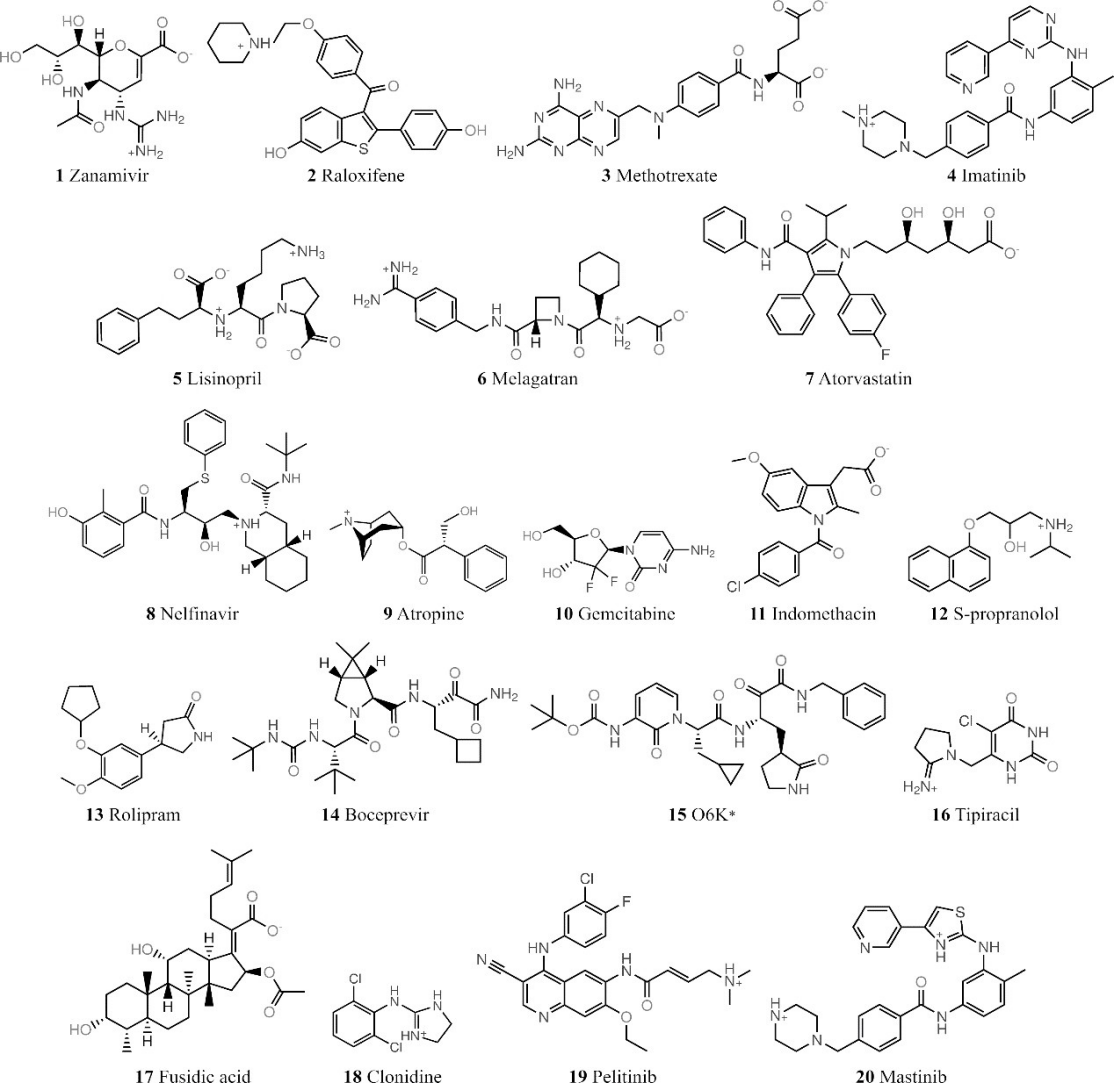
Structures of compounds in Drug20 dataset. All but the compound indicated with an asterisk are FDA‐approved.

In this work, we assess the ability of MM, QM and ML potentials to discriminate low from high energy conformations of 20 druglike molecules. The reference dataset compiled in this work, denoted the Drug20 set (Figure 1), samples molecules of diverse chemistry and size, some in folded and extended conformations. Structures are evaluated using the domain‐based local pair natural orbital (DLPNO) method,[^30^, ^31^] providing DLPNO‐CCSD(T) energies with extrapolation to the complete basis set (CBS) limit;[^32^, ^33^] the set provides a broad, exacting range of internal energies for a given ligand. Using the Drug20 set, we assess the performance in discriminating conformer energetics of NDDO and DFTB methods, the machine learning potentials ANI‐2x, ANI‐1ccx and MACE‐OFF23(L), the ωB97X/6‐31G* level of theory and the MMFF94 force field.^[34]^

## Computational Methods

### Reference Conformer Dataset

The reference Drug20 database compiled for this work comprised 20 druglike ligands **1**–**20** (Figure 1), 19 of which are approved therapeutics. A subset of these compounds have featured in previous conformer datasets[^7^, ^35^] although the conformations generated here are distinct, arising from the protocol outlined below. We also note that seven of the Drug20 ligands (**14**–**20**) are SARS‐CoV‐2 ligands, among which **16**–**20** are approved drugs repurposed against the SARS‐CoV‐2 main protease (M^pro^). The initial geometries were retrieved from the RCSB Protein Data Bank (PDB) in their protein‐bound pose. Table S1 provides the details of the ligands, including their PDB entry codes, and their corresponding protein targets.

For each ligand, one major protonation state was assigned with Protonate3D from the Molecular Operating Environment (MOE) package^[36]^ at neutral pH (Table S1). Exceptions were made for ligand **14** and ligand **15**, both of which are covalent inhibitors. The ketoamide groups in the covalent form was back‐transformed to a sp^2^ state centre prior to attack by the nucleophilic S atom of Cys145 in the SARS‐CoV‐2 main protease.^[37]^ The geometries around this centre were relaxed via MMFF94x^[34]^ optimisations in MOE.

To generate solution structures of the compounds **1**–**20**, a conformational search was performed via the LowModeMD^[38]^ method implemented in MOE, with an energy window of 15 kcal/mol. The MMFF94x force field was used with a generalised Born (GB) implicit solvent model. For each molecule, up to eight of the resulting solution conformations, including the lowest energy, were selected to maximise structural diversity. These structures were then optimised, using the ORCA package,[^39^, ^40^] at the PBE0‐D3BJ/def2‐TZVPP[^41^, ^42^] level with the conductor‐like polarizable continuum model (CPCM), an implicit solvent model, using a dielectric constant of 80.^[43]^ While most conformers after PBE0‐D3BJ/def2‐TZVPP/CPCM optimisation remained in a pose similar to their corresponding MMFF94x/GB generated structures, 5 % optimised to structures identical to others in the dataset. These resulting duplicate structures were manually identified and removed from the dataset (Figure S1), leaving a total of 140 unique solution conformers over molecules **1**–**20** to form the Drug20 dataset.

For these structures, single point gas‐phase energies were computed using the DLPNO‐CCSD(T) method[^30^, ^31^] with the “TightPNO” setting. This approach has been shown to recover 99.9 % of the canonical correlation energy and yields an RMSD of ~0.6 kcal/mol in relative energies compared to canonical CCSD(T) when using the same basis set.[^31^, ^44^, ^45^] To estimate the complete basis set value, the two‐point extrapolation method of Helgaker et al. was used[^32^, ^33^] with the aug‐cc‐pVDZ and aug‐cc‐pVTZ basis sets. We denote the two‐point extrapolation scheme as CBS(2,3), reflecting the cardinal numbers of the two basis sets used in extrapolation. Using a LPNO scheme, the CBS(2,3) method has been found to introduce an additional average error of ~0.1 kcal/mol relative to the corresponding CBS(3,4) calculations which extrapolate with the aug‐cc‐pVTZ and aug‐cc‐pVQZ basis sets.^[46]^ Accordingly, for each ligand, solution conformers were ranked based on their DLPNO‐CCSD(T)/CBS(2,3) estimated relative potential energies ΔE; these conformers were labelled as ***N‐m***, where ***N*** represents the compound number and ***m***, starting from 1, indicates the ranking of the compound's conformers according to ΔE, from low to high energy, for the respective ligand ***N***. The geometries and energetics of the Drug20 dataset can be downloaded from the Figshare repository at https://doi.org/10.6084/m9.figshare.27136077.v3.

### Calculation of Conformer Energetics

To assess the internal energies of the Drug20 conformers via lower level methods, single point gas‐phase energies via the approximate method were computed at the reference geometries. In addition, reference geometries were optimised using the lower level method with a solvent model. The exception was for the ML potentials, where solvent optimisations were not available for ANI or MACE‐OFF23(L) and so gas phase geometries were used. Density functional ωB97X/6‐31G*//ωB97X/6‐31G*/CPCM energies^[47]^ were obtained via the ORCA suite of programs. Tight binding density functional DFTB3,^[13]^ DFTB3‐D3,^[48]^ and DFTB3‐D3H5^[17]^ calculations were carried out using the DFTB+ software^[49]^ with the 3ob‐3‐1 parameter set.^[50]^ The geometry convergence cutoff for these methods was set so that the final force component on any atom would be below 1.0×10^−4^ eV/Å. The analytical linearised Poisson‐Boltzmann (ALPB) solvation model was employed to account for solvent effects.^[51]^ The Anderson mixing parameter was adjusted to 0.015 to enhance convergence for certain conformers. GFN1‐xTB^[14]^ and GFN2‐xTB^[15]^ calculations were conducted using the xtb program.^[52]^ The geometry optimisation was performed with the ALPB solvation model, and the convergence criterion was set to tight, which allowed the total energy change of 1.0×10^−6^ E_h_. PM6D3H4X^[53]^ and PM7^[54]^ calculations were performed with the MOPAC2016 software,^[55]^ where the geometry optimisation criterion was set to a difference of 0.002 kcal/mol between two consecutive steps. The conductor‐like screening model (COSMO) was applied during optimisation.^[56]^ ANI‐1ccx^[23]^ and ANI‐2x^[24]^ calculations were carried out with TorchANI.^[57]^ Geometry optimisation using ANI methods was performed using the conjugate gradient backtracking line search (CG‐BS) algorithm,^[58]^ which in recent studies was shown to ameliorate the issue of roughness in ANI potential energy surfaces.[^59^, ^60^] For these optimisations, the maximum and RMS force criteria were set to 0.000225 and 0.00015 au, while the maximum and RMS displacement were 0.0009 and 0.0006 au, respectively. MACE‐OFF23(L)^[28]^ calculations were conducted via the ASE interface.^[61]^ The L‐BFGS optimisation algorithm was used for MACE‐OFF23(L) calculations, and the geometry convergence criteria was set to a maximum force component of less than 0.01 eV/Å on any atom. Finally, MMFF94^[34]^ calculations were performed using the *obenergy* and *obminimize* modules implemented within the OpenBabel suite of software.^[62]^

## 2 Results and Discussion

### 2.1 Reference Drug20 Conformer Dataset

**Figure 2:**
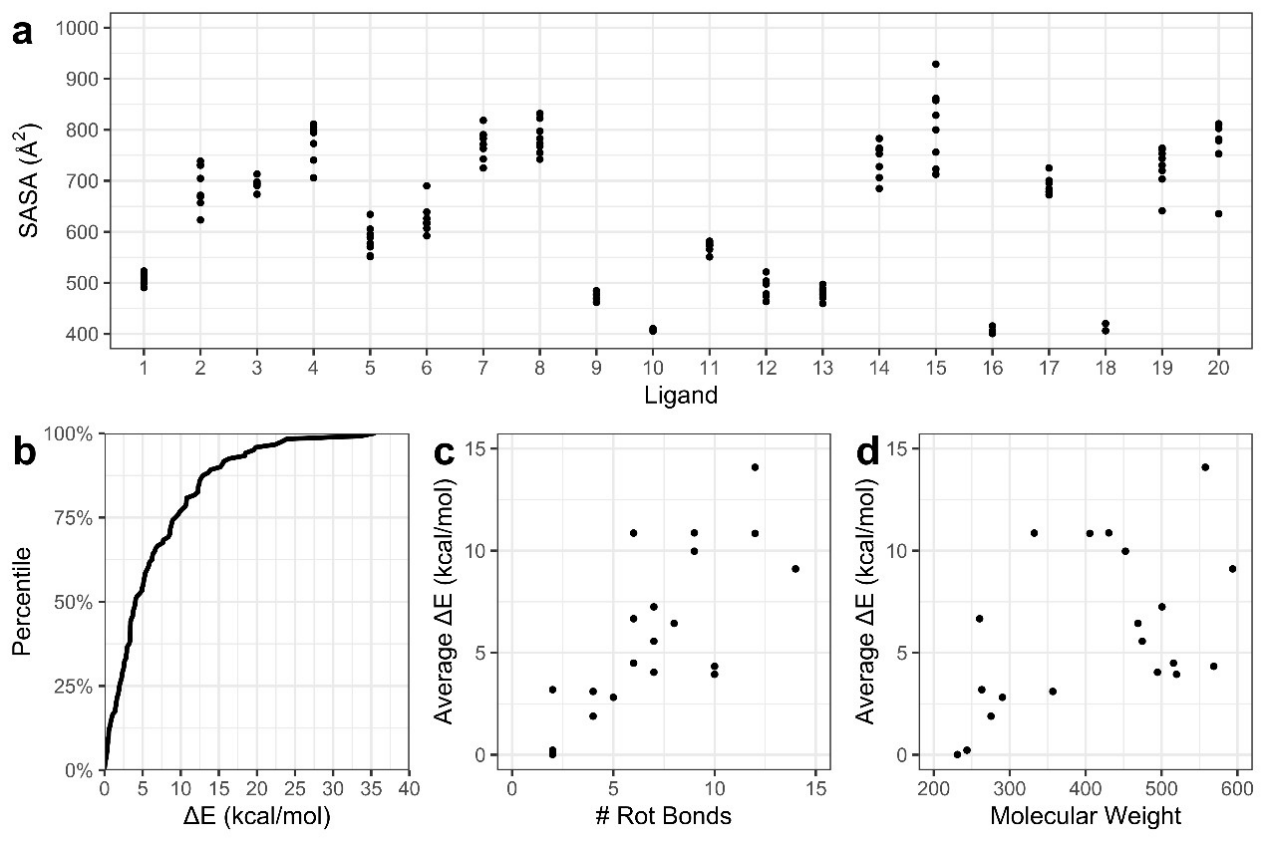
(a) Solvent‐accessible surface area (SASA) for each Drug20 conformer of ligand **1**–**20**. (b) Cumulative distribution of conformational energies. Average relative energies of ligand with respect to (c) number of rotational bonds, and (d) molecular weight (g/mol).

In order to assess the ability of MM, QM and ML potentials to discriminate low from high energy conformations of druglike molecules, we construct the Drug20 reference conformer dataset, with energies evaluated at the DLPNO‐CCSD(T)/CBS(2,3) level of theory. The solution conformer geometries were generated via LowModeMD with subsequent optimisation at the PBE0‐D3BJ/def2‐TZVPP/CPCM level; these structures exhibit sampling of compact and extended structures, reflected by the range in solvent‐accessible surface area (SASA) in Figure 2a; the largest SASA range is found for molecule **15**, which also has the greatest number of rotatable single bonds in the set, namely 14 (Table S1). The diversity in conformer geometry is further reflected by the broad distribution of Cartesian RMSD values across conformers for each molecule, with an average RMSD of 2.7 Å and maximum RMSD of 4.7 Å (Figure S2).

The sampling protocol leads to a relative energy range for Drug20 molecules which is wider than that of comparable datasets, with the largest DLPNO‐CCSD(T)/CBS(2,3) energy range of 35.4 kcal/mol (for ligand **6**); this compares with a range of 14.7 kcal/mol at the PBE‐D3BJ/def2‐TZVP level for the 37Conf8 set;^[7]^ and 25.1 kcal/mol at the DLPNO‐CCSD(T)/cc‐pVTZ level for Folmsbee and Hutchison's dataset.^[29]^ Nevertheless, the majority of Drug20 conformers have low relative energies, with 62 % of conformers’ ΔE values below 6 kcal/mol and 74 % below 9 kcal/mol of their respective global energy minimum (Figure 2b). We note that the range in ΔE for a given Drug20 molecule is somewhat related to its size, as reflected by number of rotatable bonds (Figure 2c), although with a weaker correlation to molecular weight (Figure 2d).

### 2.2 Methods Comparison with Drug20 Dataset

**Figure 3:**
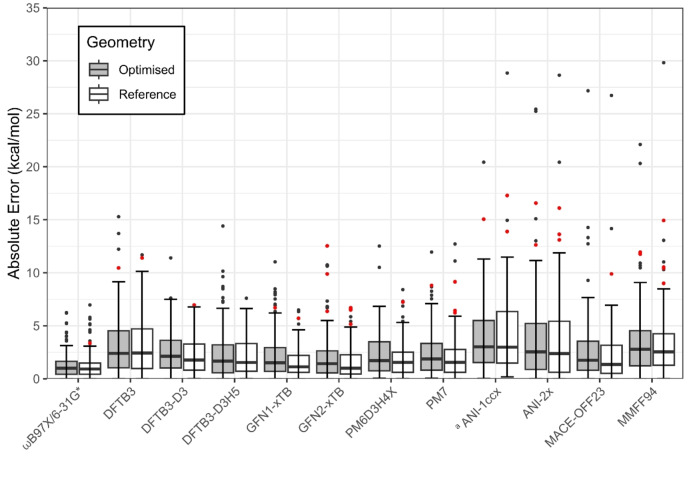
Distribution of absolute error in relative conformational energies ΔE of assessed methods with respect to DLPNO‐CCSD(T)/CBS(2,3) values of Drug20 set, at optimised and reference DFT geometries. From bottom to top of boxplot: minimum, quartile 1 (Q1), median, quartile 3 (Q3), upper boundary of data (Q3+1.5 *IQR) and outliers. Outliers which are false positive (FP) or false negative (FN) conformers for the corresponding method and geometry in red; see text for definition of FP and FN. ^[a]^ ANI‐1ccx was applied to 81 solution conformers of the 11 CHNO‐only molecules in Drug20.

We next turn to assess the ability of a range of more approximate potentials to reproduce the benchmark DLPNO‐CCSD(T)/CBS(2,3) conformational energies of the Drug20 set, using either the reference PBE0‐D3BJ/def2‐TZVPP/CPCM or method‐optimised geometries. Of the MM, ML and QM potentials evaluated, the ωB97X/6‐31G* level of theory was found to achieve the lowest mean absolute error (MAE) compared to the reference ΔE values, with a MAE of 1.3 kcal/mol using reference geometries (Figure 3, Table 1). However, only 58.3 % of its predictions were within 1 kcal/mol of the DLPNO‐CCSD(T)/CBS(2,3) value, denoted as “chemically accurate” (“%CA” in Table 1). Upon optimisation at the ωB97X/6‐31G*/CPCM level, the MAE in ΔE increased by 0.1 kcal/mol, and the %CA decreased slightly to 50.0 % (Figure 3, Table 1).

**Table 1:** For each method at reference geometries and in parentheses at optimised geometries, the percentage of predictions within chemical accuracy (±1 kcal/mol) %CA, mean absolute error (MAE) in kcal/mol, squared Pearson correlation coefficient *r* ^*2*^ and Kendall's τ coefficient calculated between the conformational energies predicted by the reference and target method. The average Cartesian RMSD in Å between the reference PBE0‐D3BJ/def2‐TZVPP/CPCM structures and each assessed method. ^a^ ANI‐1ccx was applied to 81 solution conformers of the 11 CHNO‐only molecules in Drug20.

Method	%CA	MAE	*r* ^*2*^	Kendall's τ	RMSD
ωB97X/6‐31G*	58.3 (50.0)	1.3 (1.4)	0.96 (0.95)	0.82 (0.83)	0.18
DFTB3	25.8 (25.0)	3.1 (3.2)	0.74 (0.73)	0.53 (0.56)	0.58
DFTB3‐D3	30.8 (24.2)	2.2 (2.5)	0.85 (0.79)	0.65 (0.62)	0.43
DFTB3‐D3H5	36.7 (36.7)	2.1 (2.4)	0.87 (0.76)	0.66 (0.61)	0.46
GFN1‐xTB	42.5 (35.0)	1.6 (2.2)	0.91 (0.82)	0.73 (0.65)	0.41
GFN2‐xTB	50.0 (41.7)	1.6 (2.2)	0.88 (0.78)	0.76 (0.63)	0.42
PM6D3H4X	39.2 (35.8)	1.9 (2.3)	0.86 (0.83)	0.70 (0.66)	0.34
PM7	39.2 (32.5)	2.2 (2.4)	0.80 (0.83)	0.67 (0.70)	0.33
MMFF94	17.5 (21.7)	3.6 (3.6)	0.59 (0.59)	0.55 (0.51)	0.49
ANI‐1ccx^[a]^	17.1 (17.1)	4.6 (4.1)	0.31 (0.48)	0.36 (0.32)	0.19
ANI‐2x	30.0 (30.0)	3.7 (3.8)	0.38 (0.38)	0.46 (0.47)	0.24
MACE‐OFF23(L)	38.3 (30.0)	2.3 (2.7)	0.69 (0.66)	0.67 (0.65)	0.21

Several NDDO‐based and tight‐binding density functional SQM methods were evaluated, with MAEs in relative energy ranging from 1.6 to 3.1 kcal/mol (Table 1, Figure 3). Addition of a post‐SCF correction was found to significantly improve the accuracy of DFTB3, such that DFTB3‐D3H5 gave an improvement of 1 kcal/mol in MAE in ΔE (Table 1); this was achieved without compromising computational efficiency. Among the SQM methods examined here, the GFN2‐xTB method yielded the highest chemical accuracy, with %CA values of 50.0 % at reference geometries and 41.7 % at optimised geometries (Table 1). The corresponding MAE of 1.6 kcal/mol on reference geometries is similar to its predecessor GFN1‐xTB and considerably lower than the DFTB3 counterparts (Figure 3).

Accordingly, among the SQM potentials, the GFN2‐xTB method provided the most accurate ranking of conformers according to ΔE, with a Kendall's τ coefficient of 0.76 at reference geometries (Table 1). On optimisation, this correlation with the reference reduced somewhat to a value of 0.63, suggesting a difference between the GFN2‐xTB and PBE0‐D3BJ/def2‐TZVPP/CPCM energy surfaces. Despite a generally good performance, the GFN2‐xTB method was found to predict high errors in ΔE for methotrexate (**3**, Figure 1). The global minimum conformer's relative energy was overestimated by ~6 kcal/mol by all DFTB‐based methods, indicated by MAE values ranging from 7.1 kcal/mol via DFTB3 to 4.9 kcal/mol via DFTB3‐D3H5 (Table S2).

With regard to machine learning potentials, application of the ANI‐2x method to the Drug20 geometries yielded a MAE in ΔE of 3.7 kcal/mol, a %CA of 30.0 % and Kendall's τ coefficient of 0.46 (Table 1). These values did not change significantly on gas‐phase optimisation via the ANI‐2x model (Table 1). The absolute error range of ANI‐2x for both reference and optimised geometries showed significant dispersion, somewhat similar to the MMFF94 force field, the latter exhibiting a MAE in ΔE of 3.6 kcal/mol (Table 1, Figure 3). The ANI‐1ccx model, ^[20]^ trained using DLPNO‐CCSD(T)/CBS energies but only for the elements, C, H, O and N, was applied to the 81 CHNO‐only conformers of Drug20. The MAE in ΔE using ANI‐1ccx was 4.6 and 4.1 kcal/mol for Drug20 and model‐optimised geometries respectively (Table 1). The ANI‐1ccx and ANI‐2x potentials demonstrated good accuracy in reproducing PBE0‐D3BJ/def2‐TZVPP/CPCM geometries, yielding average Cartesian RMSDs from the DFT geometries of 0.19 Å and 0.24 Å, for ANI‐1ccx and ANI‐2x respectively (Table 1).

**Figure 4:**
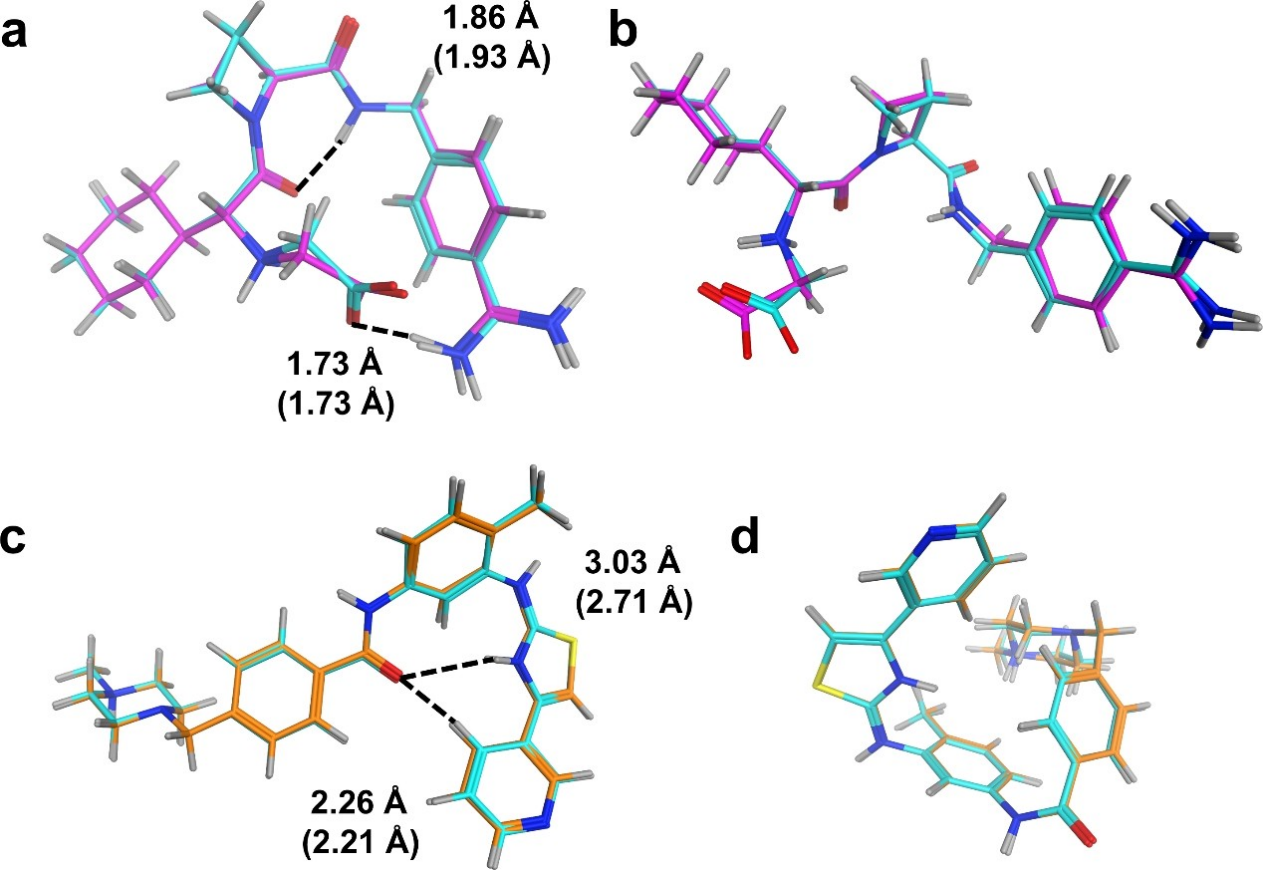
Conformers (a) **6**–**1**, (b) **6**–**7**, (c) **20**–**1** and (d) **20**–**8** with geometries generated at the reference PBE0‐D3BJ level (cyan), ANI‐2x (magenta) and MACE‐OFF23(L) (orange). Selected distances marked with dotted lines, and values of length in Å for reference geometry, and for ANI‐2x and MACE‐OFF23(L) in parentheses.

Interestingly, the ANI‐2x and in particular ANI‐1ccx methods were previously shown to be effective in computing the relative energetics of monosaccharide conformations.^[25]^ However, given the larger molecule size of the Drug20 set, the short‐range nature of the atomic environment vectors (AEVs) in the ANI models may limit their effectiveness.^[24]^ We consider the example of the thrombin inhibitor, melagatran (**6**, Figure 1), in folded (**6**–**1**, Figure 4a) and extended conformations (**6**–**7**, Figure 4b). ANI‐2x is capable of reproducing reasonable geometries for both conformers (Figure 4a,b and Table S3). However, the energy difference between the folded conformer **6**–**1**, which features an intramolecular hydrogen bond and an ionic interaction (Figure 4a), and the extended structure **6**–**7**, is underestimated by 28.6 kcal/mol (Table S4). While the inclusion of a dispersion correction may improve agreement,^[63]^ this is unlikely to address the partial accounting of the carboxylate‐amidine ion pair energy in **6**–**1** by the ANI methods.

The MACE‐OFF23(L) potential was also applied to the Drug20 conformer set. The method was found to yield MAEs of 2.3 kcal/mol on reference geometries and 2.7 kcal/mol on model‐optimised structures (Table 1), approaching the accuracy of the best‐performing SQM methods (Figure 3, Table 1). MACE‐OFF models employ a graph neural network framework and a multi‐layer message passing architecture, which collectively incorporates effects beyond nearest neighbour atoms. Consequently, we observe improved performance for the stability of **6**–**1** relative to **6**–**7**, with a ΔE of 21.2 kcal/mol (Table S4).

Nevertheless, some significant outliers were observed for the ML potential: this was particularly the case for conformer **20**–**8**, where the error in ΔE exceeds 25 kcal/mol (Figure 3, Table S4). For this molecule with five rings (Figure 1), MACE‐OFF23(L) accurately reproduced the PBE0‐D3BJ/def2‐TZVPP/CPCM reference geometries of conformers **20**–**1** and **20**–**8**, with Cartesian RMSD values of 0.15 Å and 0.18 Å, respectively (Table S3). Conformer **20**–**1** forms an extended, largely planar conformation (Figure 4c); by contrast, conformer **20**–**8** possesses a more folded conformation, where the aniline conjugation to the π‐system is partially disrupted by an out‐of‐plane rotation and an unfavourable *cis*‐amide orientation is adopted (Figure 4d). MACE‐OFF23(L) underestimates the relative energy of conformer **20**–**8** by 26.7 kcal/mol, reducing the reference ΔE of 33.6 kcal/mol predicted by DLPNO‐CCSD(T)/CBS(2,3) to 6.9 kcal/mol (Table S4). The relative energy of **20**–**8** is also underpredicted via ANI‐2x, by 20.4 kcal/mol, suggesting that neither MLP appears to describe well the extended π‐conjugation of molecule **20**. With regard to the *cis*‐amide linkage in **20**–**8**, we note that one other conformer of Drug20 featured a *cis*‐amide isomer, namely **19**–**2**. In the latter case, both ANI‐2x and MACE‐OFF23(L) successfully predicted the relative energy of **19**–**2**, with errors in ΔE of 1.1 and 2.2 kcal/mol, respectively (Table S4).

### 2.3 Discrimination of High and Low Energy Conformers

While the preceding analysis provides an overall assessment of the ability of computational potentials to rank a spectrum of conformers for a molecule, in docking or *de novo* design campaigns, it is often the approach to use a specific energy cutoff criterion to exclude high energy molecules from further study. Consequently, we next evaluate the ability of selected potentials to distinguish high energy (true negative, TN) from low energy (true positive, TP) conformers, focusing on the GFN2‐xTB, MACE‐OFF23(L) and ANI‐2x methods. The energy cutoff used is 6 kcal/mol, a criterion that follows other studies.[^35^, ^58^] False positives (FP) occur where high energy conformers are incorrectly included for further study; and false negatives (FN) define low energy conformers that are erroneously excluded.

**Figure 5:**
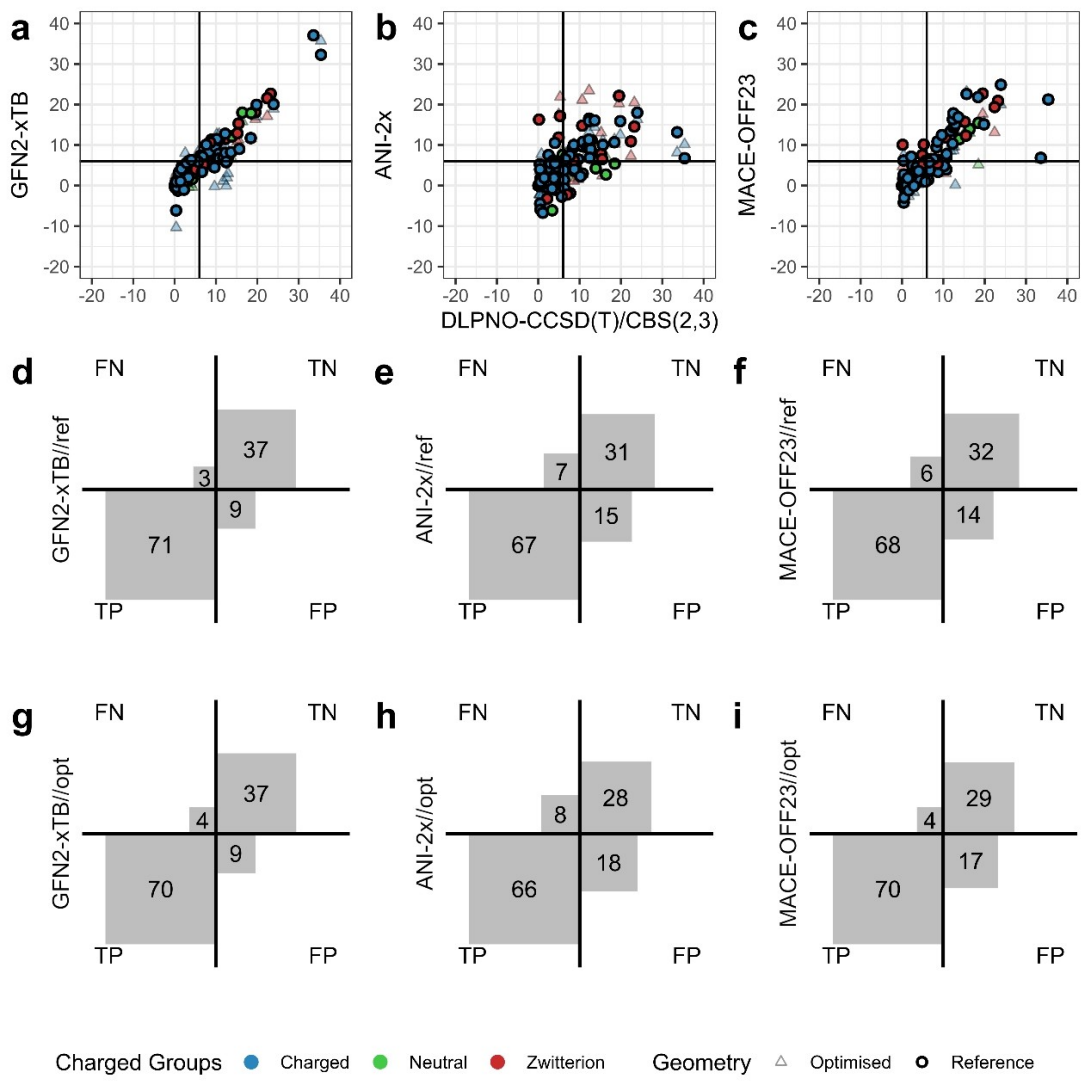
(a–c) Correlation between reference DLPNO‐CCSD(T)/CBS(2,3) conformational energies and GFN2‐xTB, ANI‐2x. MACE‐OFF23(L) predicted conformational energies, at reference DFT structures (circle) and at optimised structures (triangle), for charged molecules (blue), neutral molecules (green) and zwitterion (red). (d–f) Number of predictions falls within the defined energy quadrants at reference PBE0‐D3BJ/def2‐TZVPP/CPCM structures, and (g–i) at GFN2‐xTB, ANI‐2x, MACE‐OFF23(L) optimised structures.

We first examine the performance of the GFN2‐xTB method: as observed above, the energy predictions of GFN2‐xTB correlate well with DLPNO‐CCSD(T)/CBS(2,3) values at reference geometries, with a Kendall's τ value of 0.76 (Table 1, Figure 5a). The method exhibits a high sensitivity (TP/(TP+FN)), of 96 % (Table S5), such that 71 out of 74 lower energy conformers are correctly identified (Figure 5d). 80 positive predictions were made by GFN2‐xTB in total, resulting in a positive predictive rate (PPV; TP/(TP+FP)) of 89 % (Table S5). The ability to filter out conformers with unfavourable internal energies is less robust, with 37 out of 46 conformers correctly identified, yielding a specificity (TN/(TN+FP)) of 80 % (Table S5). On optimisation via GFN2‐xTB, 5 % of conformers collapsed to different local minima, reflected in some shifted energies in Figure 5a. Despite this, the ability of GFN2‐xTB to recognise TP and TN changes only slightly, with a sensitivity of 95 % and specificity of 80 % (Figure 5g, Table S5). Similarly, the PPV and negative predictive rate (NPV; TN/(TN+FN)) slightly decrease to 89 % and 90 %, respectively (Table S5).

For the ANI‐2x machine learning potential, a lower correlation with the reference energies at reference geometries than for GFN2‐xTB is observed (Figure 5b): for ANI‐2x, a Kendall's τ value of 0.46 and 0.47 is found at DFT and model‐optimised geometries respectively (Table 1). The corresponding MAE in relative energies is also similar, with values of 3.7 and 3.8 kcal/mol respectively (Table 1). We note the ability of ANI‐2x to reproduce PBE0‐D3BJ/def2‐TZVPP/CPCM geometries with very good accuracy, giving an average Cartesian RMSD of 0.24 Å from DFT structures (Table 1) despite the absence of a solvent model for the MLP. Perhaps unsurprisingly, given the lack of charged species in the ANI‐2x training set,^[24]^ the lowest errors in relative energy are found for neutral, non‐zwitterionic conformers, with a MAE of 2.9 and 2.1 kcal/mol at DFT and ANI‐2x geometries (Table S6). These errors rise to 3.4 and 3.6 kcal/mol for net charged species, and further increase to 7.2 and 7.9 kcal/mol for zwitterionic species (Table S6). The superior ability of ANI‐2x to model neutral molecule conformations as compared to those of charged molecules has also been noted in a recent study by Han et al.^[58]^

The ANI‐2x potential exhibits a good sensitivity, of 91 %, in selecting low energy conformers from the Drug20 set, slightly lower than that of GFN2‐xTB (Figure 5e, Table S5). For recognising strained high energy conformers, ANI‐2x achieved a specificity of only 67 %. Upon optimisation via ANI‐2x, there were slight decreases in both sensitivity and specificity (Table S5): 39 % of the unfavourable high internal energy conformers were incorrectly categorised as low energy (Figure 5h), suggesting ANI‐2x's lower accuracy in recognising high energy conformers. This issue has recently been recognised in another recent study considering the structures of aerosol clusters, formed by species such as sulfuric acid, dimethylamine and succinic acid,^[64]^ indicating the necessity for future MLPs to cover a more comprehensive molecular rotational space.^[65]^

Finally, we consider the ability of the MACE‐OFF23(L) machine learning potential to discriminate high from low energy conformers. The method exhibits a reasonable correlation to the reference energies, with a Kendall's τ value of 0.67 (Table 1), although two outliers were found in the high energy region (Figure 5c). Similar to ANI‐2x, MACE‐OFF23(L) yields the lowest MAE in relative energy for the neutral molecules of Drug20, with a value of 0.9 kcal/mol (Table S6); the associated squared Pearson's correlation coefficient is 0.99. For charged and zwitterionic molecules, the method produces higher MAEs of 2.7 and 2.6 kcal/mol, respectively. While geometry optimisation using the MACE‐OFF23(L) potential slightly increases MAEs in relative energy across all groups of molecules with different charge states, the trend of charge‐dependent performance remains evident, a dependence that is observed to a lesser degree using GFN2‐xTB (Table S6).

In terms of discriminating high from low energy conformers, MACE‐OFF23(L) yields a sensitivity of 92 % and a specificity of 70 % (Table S5). Notably, on gas‐phase optimisation via MACE‐OFF23(L), the sensitivity increases modestly, from 92 % to 95 %. This may relate to alleviation of internal energy in the stiff degrees of freedom by structural relaxation. In the high energy region, 37 % of these unfavourable conformers were incorrectly categorised (Figure 5c,i), similar to the value of 39 % found for ANI‐2x. This is despite the very good reproduction of DFT geometries by both ANI‐2x and MACE‐OFF23(L) models, with average Cartesian RMSDs of 0.24 Å and 0.21 Å, respectively (Table 1).

## 3 Conclusions

In this study, the ability of DFT, SQM, MLP and MM methods to predict accurate conformational energies for a set of druglike molecules was assessed. To enable this analysis, we compiled the Drug20 dataset, comprising FDA‐approved drug molecules and SARS‐CoV‐2‐related inhibitors. This collection of ligands covers a range of size and chemical complexity, forming a benchmark drug molecule database, with single point potential energies computed at the DLPNO‐CCSD(T)/CBS(2,3) level of theory using PBE0‐D3BJ/def2‐TZVPP/CPCM geometries.

DFT calculations at the ωB97X/6‐31G* level gave the closest agreement with the benchmark conformer energies. Of all the lower level methods, GFN2‐xTB exhibited the best overall correlation (Kendall's τ of 0.76), and highest percentage of predictions within chemical accuracy (%CA of 50.0 %). The GFN2‐xTB method was generally accurate in reproducing conformer energetics for both flexible and relatively rigid molecules, and across various molecular charge states. We note that in a benchmarking assessment of the conformations of 37 biologically relevant molecules,^[7]^ DFTB methods were similarly found to outperform MMFF94 and PMx methods in predicting accurate conformational energies, with lower mean absolute errors to reference QM values across all molecules. In another study,^[66]^ for a set of 13 peptides and macrocyclic molecules, PMx and DFTB methods were found to achieve errors in conformational energies of 3–5 kcal/mol relative to the CCSD(T)/CBS reference data. Finally, we also note the good agreement observed between GFN2‐xTB and experimental crystallographic and gas‐phase torsional preferences for a wide variety of small molecules.^[67]^

With regard to machine learning potentials, the ANI‐2x potential has previously been found to outperform conventional docking scoring functions,^[27]^ and has demonstrated good accuracy in energy predictions for monosaccharide conformations.^[25]^ However, for the Drug20 dataset, a strong correlation between ANI‐2x and reference relative energetics was not observed. A similar observation was made in a recent post‐docking ligand strain estimation study, where the conformational energy landscape of ANI‐2x showed local differences compared to the corresponding B3LYP/6‐31G** landscape.^[68]^ The Drug20 set features compounds that considerably exceed monosaccharides in terms of size and conformational flexibility, and there was evidence of issues in capturing interactions beyond the range of the AEV descriptors in the ANI model. Furthermore, the Drug20 ligands possess a diversity of charge states, which again proved challenging for the ANI models, potentially arising from a lack of charged molecules in the ANI training dataset.

The MACE‐OFF23(L) method, another neural network potential, exhibited a good correlation with reference ΔE values, giving a MAE comparable to PM7 for this dataset. As with ANI‐2x, MACE‐OFF23(L) reproduces reference DFT structures well. However, the MLP shared comparable challenges to ANI‐2x in modelling some highly unfavourable conformers with complex electronic structure, likely due to lack of similarly high energy molecules in the training dataset. The multi‐layer message passing architecture of the MACE‐OFF23(L) model appears to capture longer range interactions reasonably effectively, with the molecular sizes of the Drug20 set lying within the perceptive field of the model. Some outliers however did also exist in the high internal energy region. Furthermore, geometry and energy calculations using MACE‐OFF23(L) did appear to run more slowly than for ANI‐2x and GFN2‐xTB, which is an additional factor to consider when addressing high‐throughput tasks such as filtering large molecular databases during a virtual screen.

Indeed, for GFN2‐xTB, ANI‐2x and MACE‐OFF23(L), the methods proved more effective at identifying low energy conformers rather than unfavourable ones. This would impact the effectiveness of *de novo* design or virtual screening campaigns if these methods were used as internal energy filters, where high energy FP conformers would potentially compete with low energy TP conformers. However, to enrich the training of ANI‐2x and MACE‐OFF23(L) potentials with a greater diversity of high energy conformers may require significant computational investment. The GFN2‐xTB method produced the lowest number of false positives and appears currently the most suitable of the three methods to act as a filter for integration into drug discovery workflows.

## Conflict of Interests

The authors declare no conflict of interest.

4

## Supporting information

As a service to our authors and readers, this journal provides supporting information supplied by the authors. Such materials are peer reviewed and may be re‐organized for online delivery, but are not copy‐edited or typeset. Technical support issues arising from supporting information (other than missing files) should be addressed to the authors.

Supporting Information

## Data Availability

The data that support the findings of this study are openly available in the Figshare repository with the identifier https://doi.org/10.6084/m9.figshare.27136077.v3.
