# Supplementary material for: Discriminating High from Low Energy Conformers of Druglike Molecules: An Assessment of Machine Learning Potentials and Quantum Chemical Methods
Source: Chemphyschem. 2025 Feb 27;26(8):e202400992. doi: 10.1002/cphc.202400992 (PMC12005129; doi:10.1002/cphc.202400992)
Supplement: Supplementary file 1 — Supporting Information [file CPHC-26-e202400992-s001.pdf]

ChemPhysChem

Supporting Information

Discriminating High from Low Energy Conformers of Druglike Molecules: An Assessment of Machine Learning Potentials and Quantum Chemical Methods

Linghan Kong and Richard A. Bryce*

Discriminating high from low strain conformers of druglike molecules: an assessment of machine learning potentials and quantum chemical methods

Linghan Kong and Richard A. Bryce†*

† Division of Pharmacy and Optometry, School of Health Sciences, Manchester Academic Health Sciences Centre, University of Manchester, Oxford Road, M13 9PT, UK

Corresponding Author

*Richard Bryce, Division of Pharmacy and Optometry, School of Health Sciences, University of Manchester, Manchester, M13 9PT, U.K. Email: R.A.Bryce@manchester.ac.uk, Tel: (0)161-275-8345, Fax: (0)161-275-2481; ORCID 0000-0002-8145-2345

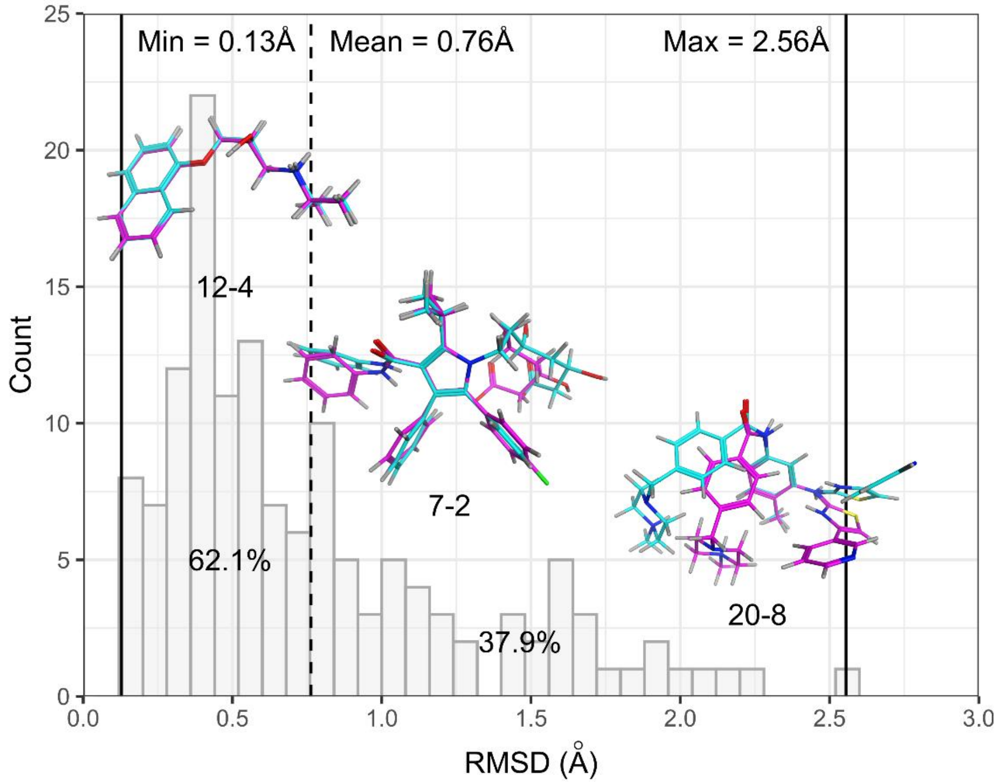

Figure S1 Distribution of Cartesian RMSD values computed from pairwise comparison of initial MMFF94x/GB generated conformer via LowModeMD in MOE with its corresponding structure after PBE0-D3BJ/def2-TZVPP/CPCM optimisation. Example structures have been provided for the conformers with the lowest, mean, and highest RMSD values, depicted with MMFF94x geometries in cyan and PBE0-D3BJ/def2-TZVPP/CPCM geometries in magenta. Conformer **20-8** underwent the most significant structural shift upon DFT optimisation, with an all-atom RMSD of 2.56 Å from its MMFF94x geometry.

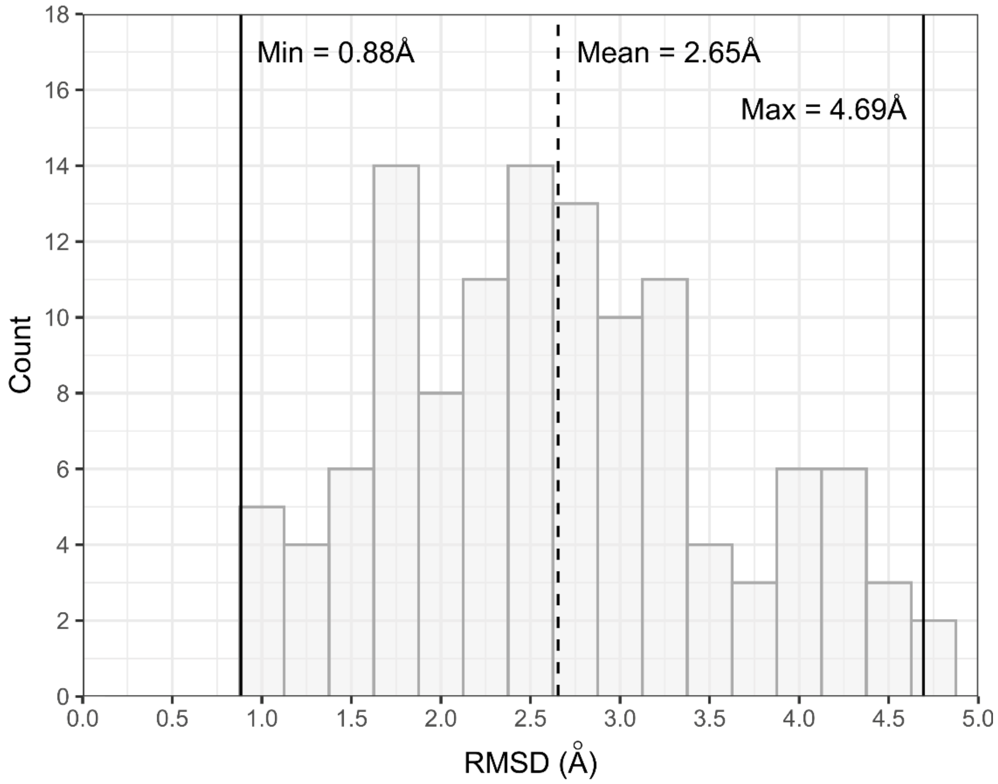

Figure S2 Distribution of Drug20 conformer Cartesian RMSD relative to their respective minimum-energy conformers. Conformers computed at PBE0-D3BJ/def2-TZVPP/CPCM geometry, based on LowModeMD conformational search with MMFF94x/GB potential.

Table S1 Ligand information of the Drug20 dataset. *An α-ketoamide inhibitor binds to the SARS-CoV-2 M^{pro}.

index	ligand name	PDB code	res (Å)	net charge	#heavy atom	#rot bond	Mol weight	Binding protein
**1**	Zanamivir	3SAN	1.60	0	23	6	332.3	Influenza A virus neuraminidase N5
**2**	Raloxifene	1QKN	2.25	1	34	7	474.6	Rat estrogen receptor beta
**3**	Methotrexate	3DAU	1.50	-2	33	9	452.4	Escherichia coli dihydrofolate reductase
**4**	Imatinib	3GVU	2.05	1	37	7	494.6	Human ABL2
**5**	Lisinopril	1O86	2.00	0	29	12	405.5	Human angiotensin converting enzyme
**6**	Melagatran	4BAH	1.94	1	31	9	430.5	Thrombin
**7**	Atorvastatin	1HWK	2.22	-1	41	12	557.6	Human HMG-CoA reductase
**8**	Nelfinavir	1OHR	2.10	1	40	10	568.8	HIV-1 protease
**9**	Atropine	2X8P	2.27	1	21	5	290.4	Choline-binding protein CbpF
**10**	Gemcitabine	2VPP	2.20	0	18	2	263.2	Drosophila melanogaster deoxyribonucleoside kinase
**11**	Indomethacin	3H1X	1.40	-1	25	4	356.8	Phospholipase A_2
**12**	S- Propranolol	1DY4	1.90	1	19	6	260.4	Cellobiohydrolase Cel7A
**13**	Rolipram	1RO6	2.00	0	20	4	275.3	Phosphodiesterase 4B
**14**	Boceprevir	6WNP	1.44	0	37	10	519.7	SARS-CoV-2 main protease
**15**	O6K*	6Y2G	2.20	0	43	14	593.7	SARS-CoV-2 main protease
**16**	Tipiracil	6WXC	1.85	1	16	2	243.7	SARS-CoV-2 Nsp15
**17**	Fusidic Acid	7A1U	1.67	-1	37	6	515.7	SARS-CoV-2 main protease
**18**	Clonidine	7AWW	1.65	1	14	2	231.1	SARS-CoV-2 main protease
**19**	Pelitinib	7AXM	1.40	1	33	8	468.9	SARS-CoV-2 main protease
**20**	Mastinib	7JU7	1.60	2	36	7	500.7	SARS-CoV-2 main protease

Table S2 Relative energies in kcal/mol at reference and at optimised geometries (in parentheses) via reference method, DFTB3, DFTB3-D3, DFTB3-D3H5, GFN1-xTB and GFN2-xTB, for molecule **3**.

Conformer	Reference	DFTB3	DFTB3-D3	DFTB3-D3H5	GFN1-xTB	GFN2-xTB
**3-1**	0.0	0.0 (0.0)	0.0 (0.0)	0.0 (0.0)	0.0 (0.0)	0.0 (0.0)
**3-2**	0.4	-7.9 (-2.3)	-6.3 (-1.4)	-6.1 (0.1)	-6.1 (-5.4)	-6.1 (-10.3)
**3-3**	9.7	3.8 (6.0)	4.5 (5.0)	5.1 (6.5)	3.9 (3.0)	4.5 (-0.2)
**3-4**	11.8	5.7 (8.9)	6.4 (8.2)	8.1 (9.0)	7.2 (7.7)	6.9 (1.2)
**3-5**	12.5	4.6 (8.4)	5.5 (7.0)	7.1 (8.7)	6.1 (4.9)	5.8 (-0.1)
**3-6**	12.7	4.8 (8.6)	5.9 (7.9)	7.5 (12.0)	6.3 (5.7)	6.2 (1.9)
**3-7**	12.9	6.2 (8.0)	7.3 (9.5)	8.6 (10.0)	7.7 (4.4)	7.1 (3.0)
MAE	0.0	7.1 (3.7)	6.1 (4.0)	4.9 (2.3)	5.8 (6.6)	5.9 (10.7)

Table S3 The cartesian RMSD in Å between the reference PBE0-D3BJ/def2-TZVPP/CPCM structures and GFN2-xTB, ANI-2x and MACE-OFF23(L), for selected conformers.

Conformer	GFN2-xTB	ANI-2x	MACE-OFF23(L)
**3-1**	1.00	0.13	0.23
**3-2**	0.29	0.18	0.14
**3-3**	0.76	0.41	0.25
**3-4**	0.41	0.15	0.37
**3-5**	0.41	0.22	0.45
**3-6**	1.31	0.25	0.35
**3-7**	0.43	0.53	1.19
**6-1**	0.20	0.31	0.50
**6-7**	0.85	0.36	0.18
**19-1**	0.17	0.16	0.16
**19-2**	0.61	0.15	0.29
**20-1**	0.35	0.06	0.15
**20-8**	0.87	0.26	0.18

Table S4 Relative energies in kcal/mol at reference and at optimised geometries (in parentheses) via reference method, GFN2-xTB, ANI-2x and MACE-OFF23(L), for selected conformers.

Conformer	Reference	GFN2-xTB	ANI-2x	MACE-OFF23(L)
**3-1**	0.0	0.0 (0.0)	0.0 (0.0)	0.0 (0.0)
**3-2**	0.4	-6.1 (-10.3)	5.2 (3.6)	6.2 (7.5)
**3-3**	9.7	4.5 (-0.2)	6.5 (1.6)	12.5 (11.5)
**3-4**	11.8	6.9 (1.2)	9.4 (8.0)	13.5 (8.5)
**3-5**	12.5	5.8 (-0.1)	9.4 (7.7)	14.3 (8.6)
**3-6**	12.7	6.2 (1.9)	9.0 (7.7)	14.6 (9.6)
**3-7**	12.9	7.1 (3.0)	11.2 (6.5)	15.1 (0.2)
**6-1**	0.0	0.0 (0.0)	0.0 (0.0)	0.0 (0.0)
**6-7**	35.4	32.3 (35.7)	6.8 (10.2)	21.2 (21.1)
**19-1**	0.0	0.0 (0.0)	0.0 (0.0)	0.0 (0.0)
**19-2**	2.6	5.2 (8.0)	3.7 (3.0)	4.8 (4.5)
**20-1**	0.0	0.0 (0.0)	0.0 (0.0)	0.0 (0.0)
**20-8**	33.6	37.1 (36.9)	13.2 (8.1)	6.9 (6.4)

Table S5 Sensitivity, specificity, positive predictive value (PPV) and negative predictive value (NPV) of GFN2-xTB, ANI-2x and MACE-OFF23(L) in distinguishing high energy conformers from low energy conformers, at reference geometries and in parenthesis at optimised geometries. The metrics were calculated as follows: sensitivity (TP/(TP + FN)), specificity (TN/(TN + FP)), PPV (TP/(TP + FP)), and NPV (TN/(TN + FN)). TP, FN, TN, and FP denote true positives, false negatives, true negatives, and false positives, respectively.

**Method**	**Sensitivity**	**Specificity**	**PPV**	**NPV**
GFN2-xTB	96% (95%)	80% (80%)	89% (89%)	93% (90%)
ANI-2x	91% (89%)	67% (61%)	82% (79%)	82% (78%)
MACE-OFF23(L)	92% (95%)	70% (63%)	83% (80%)	84% (88%)

Table S6 Squared Pearson correlation coefficient r^2 and mean absolute error (MAE) in relative energy (in kcal/mol), via GFN2-xTB, ANI-2x and MACE-OFF23(L) for DFT reference (ref) and method optimised (opt) geometries, respectively, for charged (Ch), neutral (Nt) and zwitterionic (Zw) molecules.

**Method**	r^2			**MAE**		
	**Ch**	**Nt**	**Zw**	**Ch**	**Nt**	**Zw**
GFN2-xTB//ref	0.85	0.90	0.98	1.9	1.2	1.0
GFN2-xTB//opt	0.74	0.81	0.92	2.4	1.4	2.2
ANI-2x//ref	0.47	0.11	0.11	3.4	2.9	7.2
ANI-2x//opt	0.48	0.57	0.13	3.6	2.1	7.9
MACE-OFF23(L)//ref	0.62	0.99	0.76	2.7	0.9	2.6
MACE-OFF23(L)//opt	0.60	0.71	0.78	3.0	1.4	2.9
No. Conformers	79	27	14	79	27	14

Table S7 Relative energies of Drug20 dataset in kcal/mol at reference PBE0-D3BJ/def2-TZVPP/CPCM geometries via assessed methods. ANI-1ccx was applied to the 11 CHNO-only molecules in Drug20.

Conformer	DLPNO-CCSD(T) (CBS(2,3))	PBE0-D3 (def2-TZVPP)	wB97X (6-31G*)	DFTB3	DFTB3-D3	DFTB3-D3H5	GFN1-xTB	GFN2-xTB	PM6D3H4X	PM7	ANI-1ccx	ANI-2x	MACE-OFF23(L)	MMFF94
**1-1**	0.0	0.0	0.0	0.0	0.0	0.0	0.0	0.0	0.0	0.0	0.0	0.0	0.0	0.0
**1-2**	0.2	0.9	4.7	2.0	1.8	4.8	4.6	-0.5	1.3	-1.8	1.0	16.3	10.1	-5.4
**1-3**	4.9	5.6	7.5	6.7	7.1	8.5	6.2	4.4	4.7	5.5	4.0	11.9	7.5	1.3
**1-4**	5.3	6.0	8.7	8.6	8.8	11.5	7.0	5.4	3.6	3.9	6.9	17.2	10.1	1.4
**1-5**	10.6	11.5	14.0	11.4	11.7	13.6	11.9	11.3	13.0	13.3	5.5	14.8	11.9	9.3
**1-6**	12.3	13.3	18.1	11.2	12.3	13.4	11.3	11.5	14.4	11.9	5.0	16.5	13.6	9.8
**1-7**	19.5	20.3	25.2	15.7	16.7	19.0	19.7	18.1	25.0	20.9	8.0	22.1	22.7	16.5
**1-8**	23.2	24.3	30.2	18.0	19.9	22.7	24.3	22.7	30.1	23.2	6.0	14.6	20.9	26.6
**2-1**	0.0	0.0	0.0	0.0	0.0	0.0	0.0	0.0	0.0	0.0		0.0	0.0	0.0
**2-2**	0.6	0.9	1.0	0.2	0.7	0.5	0.5	0.8	4.1	3.2		0.1	-0.7	-0.5
**2-3**	1.9	5.1	1.3	-2.7	-3.2	-4.1	0.5	2.0	-3.9	-0.6		3.1	2.0	4.4
**2-4**	2.6	2.7	2.5	-0.8	1.9	1.6	2.7	2.5	6.1	6.2		0.7	1.3	1.0
**2-5**	3.5	3.2	4.1	3.5	2.4	3.1	4.3	3.8	3.1	3.5		6.1	4.5	9.6
**2-6**	8.7	7.0	6.0	0.8	4.8	4.3	5.4	5.3	8.3	9.4		4.0	3.4	5.5
**2-7**	10.7	10.6	9.7	3.5	7.6	7.2	8.1	7.9	10.2	12.0		9.6	6.9	9.2
**2-8**	10.8	10.5	9.4	4.0	7.1	6.9	6.7	6.7	7.6	10.5		10.8	7.8	9.1
**3-1**	0.0	0.0	0.0	0.0	0.0	0.0	0.0	0.0	0.0	0.0	0.0	0.0	0.0	0.0
**3-2**	0.4	-0.7	1.7	-7.9	-6.3	-6.1	-6.1	-6.1	-1.3	-4.8	1.7	5.2	6.2	-3.0
**3-3**	9.7	8.3	11.0	3.8	4.5	5.1	3.9	4.5	11.7	8.6	3.4	6.5	12.5	17.9
**3-4**	11.8	11.8	14.6	5.7	6.4	8.1	7.2	6.9	10.2	9.6	8.8	9.4	13.5	7.6
**3-5**	12.5	12.1	15.3	4.6	5.5	7.1	6.1	5.8	10.8	9.5	9.2	9.4	14.3	7.0
**3-6**	12.7	12.6	15.4	4.8	5.9	7.5	6.3	6.2	11.2	10.1	8.9	9.0	14.6	8.7
**3-7**	12.9	12.6	16.3	6.2	7.3	8.6	7.7	7.1	12.0	10.3	8.7	11.2	15.1	11.3
**4-1**	0.0	0.0	0.0	0.0	0.0	0.0	0.0	0.0	0.0	0.0	0.0	0.0	0.0	0.0

**4-2**	0.6	0.2	1.4	-0.2	-0.7	-0.5	-0.5	-0.4	0.3	-0.4	1.6	0.9	0.5	2.0
**4-3**	2.4	2.0	1.4	-1.0	1.1	1.0	1.6	1.6	1.9	3.1	0.9	1.2	1.2	2.3
**4-4**	3.8	3.3	2.9	-0.4	1.7	1.5	2.3	2.9	3.1	3.9	1.6	1.8	1.8	3.6
**4-5**	4.6	4.4	3.9	0.0	1.4	0.4	2.1	3.2	0.5	3.1	5.3	4.5	3.6	6.5
**4-6**	6.2	7.1	5.7	5.2	7.0	7.1	6.0	7.4	4.2	6.1	2.3	3.8	3.5	10.5
**4-7**	6.7	6.7	6.2	2.1	3.7	3.3	4.5	7.1	5.6	7.5	6.0	6.9	3.8	8.0
**5-1**	0.0	0.0	0.0	0.0	0.0	0.0	0.0	0.0	0.0	0.0	0.0	0.0	0.0	0.0
**5-2**	2.1	1.9	1.9	2.3	1.8	2.0	0.9	0.6	2.0	1.0	-2.9	-3.2	1.9	3.9
**5-3**	5.1	4.8	5.4	4.2	3.3	3.6	2.9	4.0	6.1	3.6	1.8	5.9	5.4	6.2
**5-4**	7.0	7.0	6.4	3.5	6.4	5.5	4.9	5.2	8.3	7.7	0.4	-2.2	6.6	7.8
**5-5**	8.6	8.5	10.0	12.2	11.2	11.1	9.6	10.6	9.3	9.4	-0.6	6.2	5.4	17.6
**5-6**	15.2	15.4	17.1	13.1	12.0	11.4	12.1	12.9	11.3	11.6	1.3	7.8	15.7	18.6
**5-7**	15.5	15.0	16.0	14.8	16.1	15.9	13.8	15.3	13.7	14.9	4.4	6.5	12.3	22.4
**5-8**	22.4	22.6	25.5	20.9	21.4	20.2	21.4	21.6	20.1	19.7	7.5	10.9	19.4	25.5
**6-1**	0.0	0.0	0.0	0.0	0.0	0.0	0.0	0.0	0.0	0.0	0.0	0.0	0.0	0.0
**6-2**	0.4	-0.7	-0.6	3.2	3.4	3.0	2.9	1.1	2.7	5.7	-0.5	-4.5	-4.2	4.7
**6-3**	0.5	-0.5	-0.4	3.1	3.4	3.3	2.9	1.0	3.0	5.5	-0.9	-5.9	-3.0	4.1
**6-4**	3.3	2.3	1.8	1.5	1.1	0.2	1.4	2.0	2.4	4.1	-3.1	-0.8	3.4	2.5
**6-5**	5.7	4.9	5.9	8.9	8.9	9.2	8.6	5.9	8.0	12.1	1.3	-2.7	1.0	9.0
**6-6**	19.8	19.6	21.3	18.4	18.2	18.2	17.6	19.9	21.6	24.6	13.5	15.9	15.1	28.3
**6-7**	35.4	34.3	38.1	37.6	41.0	43.0	33.8	32.3	36.8	41.9	6.6	6.8	21.2	42.9
**7-1**	0.0	0.0	0.0	0.0	0.0	0.0	0.0	0.0	0.0	0.0		0.0	0.0	0.0
**7-2**	2.3	1.9	3.2	-2.7	-1.3	1.5	1.0	-1.0	3.9	2.0		4.3	7.1	1.0
**7-3**	12.3	12.8	16.7	8.5	10.3	13.0	10.6	8.1	11.1	9.6		15.9	16.3	6.4
**7-4**	12.4	11.8	13.8	5.4	8.8	11.3	10.2	5.9	8.4	8.4		6.9	17.8	7.9
**7-5**	13.6	13.4	17.2	5.3	9.2	12.5	9.2	8.2	13.2	10.3		16.1	16.9	6.4
**7-6**	15.6	15.1	18.9	5.5	12.0	14.1	13.8	9.0	13.3	10.7		10.0	22.6	0.7
**7-7**	18.4	18.5	23.6	10.4	14.6	16.5	15.6	11.7	16.6	14.3		10.7	21.8	7.4

**7-8**	23.9	24.0	29.0	17.9	22.1	23.9	23.9	20.1	20.0	18.0		18.0	24.9	13.6
**8-1**	0.0	0.0	0.0	0.0	0.0	0.0	0.0	0.0	0.0	0.0		0.0	0.0	0.0
**8-2**	0.8	0.9	-0.1	-2.4	2.1	2.3	0.8	1.5	3.9	3.6		3.6	1.5	4.3
**8-3**	1.1	1.2	2.3	4.4	2.7	1.7	1.7	3.3	-1.5	-3.6		-6.7	-0.1	8.8
**8-4**	1.6	2.7	3.8	5.8	6.0	5.1	3.9	4.1	5.3	4.2		1.9	-1.2	10.6
**8-5**	2.9	3.4	4.6	4.7	7.9	6.2	4.6	5.6	6.3	6.9		5.5	-0.1	13.3
**8-6**	4.0	4.8	5.3	4.8	9.5	8.6	4.4	6.3	7.3	7.1		5.5	3.6	8.3
**8-7**	7.7	8.6	9.9	11.3	12.9	12.9	8.0	10.1	8.3	9.1		7.4	6.6	11.5
**8-8**	12.2	13.0	13.8	9.6	14.5	14.3	9.3	12.8	10.3	12.4		11.4	10.4	9.8
**9-1**	0.0	0.0	0.0	0.0	0.0	0.0	0.0	0.0	0.0	0.0	0.0	0.0	0.0	0.0
**9-2**	0.4	0.6	1.0	0.3	0.5	1.3	1.6	0.9	2.6	2.3	2.3	4.0	2.7	-1.8
**9-3**	1.4	1.4	2.0	1.2	1.1	1.7	1.5	1.1	0.9	0.8	0.6	1.9	1.8	-1.8
**9-4**	6.6	6.5	6.9	3.2	4.3	5.1	3.4	3.4	4.0	4.8	3.9	6.5	5.7	3.0
**10-1**	0.0	0.0	0.0	0.0	0.0	0.0	0.0	0.0	0.0	0.0		0.0	0.0	0.0
**10-2**	1.8	1.3	1.2	1.5	1.3	0.7	2.5	4.9	2.1	2.0		1.6	1.2	0.2
**10-3**	2.3	2.0	1.0	-0.8	-0.8	-1.3	1.2	3.7	1.3	0.4		2.5	1.5	1.5
**10-4**	2.5	2.5	2.4	0.2	0.2	0.3	1.3	2.0	0.7	1.3		1.4	2.2	2.7
**10-5**	3.0	2.2	3.3	0.8	1.2	0.6	2.8	3.1	1.7	1.4		3.1	2.1	3.9
**10-6**	4.1	3.7	3.2	1.8	1.9	0.8	3.6	5.6	4.0	4.2		1.5	3.0	1.5
**10-7**	5.5	4.8	5.3	3.5	3.5	2.6	2.6	4.2	1.8	3.2		4.2	4.6	4.0
**11-1**	0.0	0.0	0.0	0.0	0.0	0.0	0.0	0.0	0.0	0.0		0.0	0.0	0.0
**11-2**	2.0	2.0	2.2	1.6	1.8	1.7	2.5	1.5	2.7	2.7		1.9	2.6	3.7
**11-3**	2.0	1.9	2.6	1.6	1.7	1.7	1.8	1.3	2.8	2.5		1.6	2.2	3.8
**11-4**	3.2	2.9	3.4	1.8	2.1	2.2	3.0	2.2	3.8	3.4		3.7	4.0	5.4
**11-5**	3.4	3.3	4.1	2.8	3.0	3.2	3.4	3.3	4.2	3.8		2.2	3.1	5.7
**11-6**	4.9	4.7	5.5	3.7	4.0	4.1	4.2	3.9	6.2	5.6		3.7	3.8	8.3
**12-1**	0.0	0.0	0.0	0.0	0.0	0.0	0.0	0.0	0.0	0.0	0.0	0.0	0.0	0.0
**12-2**	0.9	1.2	0.4	2.0	2.6	1.6	0.4	1.7	-0.8	-0.1	1.6	0.3	1.3	-0.6

**12-3**	3.9	2.6	2.1	2.3	3.2	2.3	0.7	3.3	0.8	1.7	0.2	-1.0	0.9	2.8
**12-4**	6.3	5.8	5.4	4.7	7.1	6.5	4.6	6.0	6.6	6.6	-1.4	1.4	2.9	4.1
**12-5**	8.6	8.5	8.0	9.2	11.5	10.9	9.3	10.7	10.2	10.4	1.3	3.7	6.3	7.3
**12-6**	8.8	8.8	8.3	9.1	11.9	11.5	9.6	10.8	10.8	10.8	0.2	4.7	5.4	5.0
**12-7**	8.9	8.9	8.0	9.8	12.3	12.0	9.8	11.3	11.1	11.1	0.0	4.9	5.5	5.6
**12-8**	9.3	9.3	9.0	9.6	12.3	12.0	10.0	11.3	11.4	11.0	0.7	6.1	5.9	5.8
**13-1**	0.0	0.0	0.0	0.0	0.0	0.0	0.0	0.0	0.0	0.0	0.0	0.0	0.0	0.0
**13-2**	1.0	1.1	1.0	1.0	1.6	1.5	1.7	1.3	2.2	2.0	0.8	1.5	0.8	0.7
**13-3**	1.4	1.9	1.4	3.9	3.6	2.9	0.7	1.9	-1.8	-0.5	3.3	2.6	1.2	-0.4
**13-4**	1.5	1.5	1.4	0.5	1.3	1.4	2.6	1.9	2.4	2.2	2.0	2.0	1.2	1.0
**13-5**	1.5	1.9	0.9	4.7	4.5	3.9	0.7	2.9	-0.1	0.9	-0.1	1.3	1.5	-0.5
**13-6**	1.6	2.0	1.2	5.1	5.0	4.4	1.6	3.2	0.8	1.8	0.1	1.8	1.4	0.2
**13-7**	2.9	3.8	1.6	7.9	7.9	6.5	1.7	4.7	-1.9	0.6	1.6	2.7	2.4	-1.0
**13-8**	3.3	3.3	3.4	1.5	0.0	0.5	0.2	1.2	-1.8	-1.2	4.3	2.7	2.1	6.4
**14-1**	0.0	0.0	0.0	0.0	0.0	0.0	0.0	0.0	0.0	0.0	0.0	0.0	0.0	0.0
**14-2**	0.8	-0.3	-0.1	-5.9	-1.3	0.0	-2.1	-1.2	0.4	-1.0	-1.4	0.3	-0.8	-4.2
**14-3**	3.4	2.6	2.8	-1.0	3.3	4.3	3.3	2.6	3.3	1.2	0.4	2.1	2.4	-2.9
**14-4**	3.7	2.8	3.6	-1.5	3.8	4.6	3.3	2.4	3.4	1.0	1.4	2.5	3.1	-2.4
**14-5**	3.7	3.0	3.2	-1.6	2.3	3.3	2.8	2.6	2.1	0.2	1.0	2.9	2.6	-1.6
**14-6**	4.3	3.6	3.7	-4.5	2.6	4.5	4.9	1.6	6.1	4.7	-0.5	3.6	3.7	4.0
**14-7**	5.8	5.6	6.8	0.8	2.8	3.7	6.0	6.5	6.1	1.6	3.9	4.0	6.2	5.1
**14-8**	5.9	5.5	6.4	0.5	-0.6	-0.7	2.8	4.0	-1.3	-5.2	7.6	7.6	5.2	7.8
**15-1**	0.0	0.0	0.0	0.0	0.0	0.0	0.0	0.0	0.0	0.0	0.0	0.0	0.0	0.0
**15-2**	0.5	0.6	1.2	0.3	0.2	0.2	0.7	0.6	0.6	0.2	2.8	1.8	0.5	-0.1
**15-3**	3.4	3.3	0.0	-2.8	4.6	5.5	1.1	3.0	10.7	12.5	-0.7	-6.1	2.3	0.7
**15-4**	3.4	3.5	2.8	2.0	4.3	4.9	1.7	2.3	3.1	4.0	3.0	-0.2	2.6	0.0
**15-5**	7.7	7.7	7.6	2.0	10.0	11.3	6.3	6.8	13.0	13.9	2.4	-1.9	5.5	3.1
**15-6**	13.9	12.9	12.0	2.5	15.5	17.2	13.0	12.1	17.3	19.2	7.5	4.2	11.5	10.9

**15-7**	16.4	16.9	16.3	10.9	20.1	21.6	18.4	18.0	21.4	22.0	4.9	2.7	13.9	9.0
**15-8**	18.5	18.8	17.3	13.7	22.5	25.1	18.5	17.9	19.3	19.7	8.2	5.4	15.5	23.0
**16-1**	0.0	0.0	0.0	0.0	0.0	0.0	0.0	0.0	0.0	0.0		0.0	0.0	0.0
**16-2**	0.0	0.0	0.0	0.1	0.1	0.1	0.0	0.1	0.1	0.1		0.0	0.0	0.1
**16-3**	0.3	0.6	0.1	1.4	1.1	1.0	0.0	0.9	-2.4	-2.2		0.4	1.7	0.1
**16-4**	0.4	0.7	0.2	1.3	1.0	0.9	0.1	0.9	-2.1	-2.1		0.3	1.8	0.2
**17-1**	0.0	0.0	0.0	0.0	0.0	0.0	0.0	0.0	0.0	0.0	0.0	0.0	0.0	0.0
**17-2**	1.9	1.7	0.7	0.6	0.6	0.3	0.2	1.6	1.9	1.5	2.4	-0.4	0.6	2.9
**17-3**	2.5	3.2	3.4	2.1	3.6	3.4	3.2	3.1	4.7	4.8	3.7	7.5	2.0	4.5
**17-4**	2.8	3.0	3.9	1.9	3.3	3.2	3.6	3.2	3.9	3.1	4.8	1.5	3.4	-0.4
**17-5**	3.3	3.3	3.9	2.0	2.7	2.6	3.0	3.6	4.0	3.1	6.0	0.2	3.6	1.0
**17-6**	4.0	5.2	4.3	2.2	2.9	2.6	3.2	3.5	5.0	4.9	6.4	10.5	3.6	7.8
**17-7**	8.3	8.1	9.8	6.0	7.5	8.2	9.4	8.3	9.3	9.1	12.3	9.2	9.5	7.2
**17-8**	8.6	9.0	10.0	1.8	5.0	5.1	5.6	5.7	3.6	2.3	13.0	10.0	10.9	4.8
**18-1**	0.0	0.0	0.0	0.0	0.0	0.0	0.0	0.0	0.0	0.0		0.0	0.0	0.0
**18-2**	0.0	0.0	0.0	0.0	0.0	0.0	0.0	0.0	0.0	0.0		0.0	0.0	-29.8
**19-1**	0.0	0.0	0.0	0.0	0.0	0.0	0.0	0.0	0.0	0.0		0.0	0.0	0.0
**19-2**	2.6	2.9	3.6	6.6	3.6	4.3	6.1	5.2	1.9	-1.2		3.7	4.8	10.2
**19-3**	5.1	4.6	3.7	2.5	4.5	4.7	4.3	4.8	5.5	5.0		-1.4	1.4	3.6
**19-4**	5.2	4.7	3.8	2.5	4.6	4.7	4.3	4.8	5.5	4.8		-1.4	1.3	3.6
**19-5**	5.3	4.7	4.1	2.4	4.3	4.5	4.2	4.8	5.9	4.9		-0.8	1.4	4.2
**19-6**	6.3	4.6	4.0	2.5	4.4	4.5	4.2	4.8	5.7	4.8		-0.7	1.4	4.2
**19-7**	9.9	9.0	8.6	8.5	8.7	9.2	7.9	9.5	4.9	5.4		3.1	4.1	10.7
**19-8**	10.8	10.5	9.8	9.2	10.1	10.4	9.8	11.2	10.1	8.7		3.0	4.0	10.1
**20-1**	0.0	0.0	0.0	0.0	0.0	0.0	0.0	0.0	0.0	0.0		0.0	0.0	0.0
**20-2**	0.1	0.1	0.1	0.2	0.2	0.1	0.1	0.0	-0.2	0.0		0.1	0.0	-0.4
**20-3**	0.2	0.3	0.3	0.4	0.4	0.5	0.3	0.4	-0.1	0.1		-0.1	-0.1	1.1
**20-4**	0.4	0.5	0.7	0.8	0.7	0.8	0.8	0.7	0.7	0.7		0.2	-0.1	0.1

**20-5**	2.8	3.0	3.1	3.4	4.1	3.9	4.4	5.2	3.4	4.2		4.0	3.1	2.6
**20-6**	3.4	3.7	3.8	4.4	5.1	4.9	5.4	6.0	3.9	4.7		4.0	3.1	2.1
**20-7**	10.2	10.1	10.6	10.7	11.6	11.9	11.4	11.5	9.8	9.3		2.3	4.8	-0.3
**20-8**	33.6	34.4	38.2	45.3	37.0	37.5	35.7	37.1	25.2	20.9		13.2	6.9	20.5

Table S8 Relative energies of Drug20 dataset in kcal/mol at method optimised geometries. ANI-1ccx was applied to the 11 CHNO-only molecules in Drug20.

Conformer	wB97X (6-31G*)	DFTB3	DFTB3-D3	DFTB3-D3H5	GFN1-xTB	GFN2-xTB	PM6D3H4X	PM7	ANI-1ccx	ANI-2x	MACE-OFF23(L)	MMFF94
**1-1**	0.0	0.0	0.0	0.0	0.0	0.0	0.0	0.0	0.0	0.0	0.0	0.0
**1-2**	-3.8	-0.1	5.6	6.2	-0.2	2.5	-2.3	-2.4	1.0	4.6	4.8	-2.7
**1-3**	6.1	4.2	4.2	2.5	3.6	3.9	1.2	4.1	7.2	17.6	6.4	2.9
**1-4**	7.9	8.2	7.7	6.8	6.7	5.9	2.1	7.3	9.4	21.8	2.4	-2.7
**1-5**	13.4	11.8	12.3	12.6	11.8	10.1	0.1	6.0	7.5	21.1	2.9	-0.3
**1-6**	17.6	12.1	13.3	12.7	13.1	11.7	6.9	15.3	8.5	23.4	14.0	10.9
**1-7**	21.1	7.3	8.1	14.3	18.9	16.3	26.4	22.8	10.9	20.2	17.7	-0.8
**1-8**	29.5	18.8	20.4	20.2	25.2	22.3	26.4	24.8	8.2	20.5	20.7	24.7
**2-1**	0.0	0.0	0.0	0.0	0.0	0.0	0.0	0.0		0.0	0.0	0.0
**2-2**	0.7	-1.7	0.6	0.4	0.7	0.5	3.0	2.1		-3.0	-1.1	-1.5
**2-3**	-0.4	-1.5	-0.9	-1.2	-0.8	-0.7	2.2	1.4		0.5	-1.3	-1.9
**2-4**	2.7	-2.4	1.5	0.8	3.3	3.1	6.5	6.7		0.1	1.1	2.6
**2-5**	4.8	-1.4	2.6	3.5	5.4	5.3	4.0	3.5		6.1	4.7	7.1
**2-6**	6.2	-0.4	4.6	3.9	6.2	6.7	8.8	10.3		3.0	3.2	6.8
**2-7**	10.0	2.4	7.6	7.4	9.0	8.8	9.5	11.6		9.8	7.2	7.6
**2-8**	9.9	3.2	7.3	7.3	7.8	8.5	8.4	10.6		10.8	8.1	0.3
**3-1**	0.0	0.0	0.0	0.0	0.0	0.0	0.0	0.0	0.0	0.0	0.0	0.0
**3-2**	3.2	-2.3	-1.4	0.1	-5.4	-10.3	5.7	3.7	2.4	3.6	7.5	-0.1
**3-3**	11.2	6.0	5.0	6.5	3.0	-0.2	22.2	17.9	-0.2	1.6	11.5	2.6
**3-4**	16.1	8.9	8.2	9.0	7.7	1.2	16.6	17.4	9.4	8.0	8.5	3.0
**3-5**	15.6	8.4	7.0	8.7	4.9	-0.1	17.2	17.5	9.5	7.7	8.6	3.4
**3-6**	16.7	8.6	7.9	12.0	5.7	1.9	13.4	19.8	9.7	7.7	9.6	2.0
**3-7**	16.0	8.0	9.5	10.0	4.4	3.0	19.7	16.6	8.5	6.5	0.2	4.1
**4-1**	0.0	0.0	0.0	0.0	0.0	0.0	0.0	0.0	0.0	0.0	0.0	0.0

**4-2**	1.7	0.3	-0.2	0.1	1.0	-0.9	-1.1	-0.9	1.2	1.4	0.2	-0.8
**4-3**	1.9	0.4	1.9	1.5	2.2	1.7	0.8	2.5	1.6	3.0	1.4	2.5
**4-4**	3.1	0.8	2.3	1.9	2.6	3.0	2.9	4.1	2.6	3.5	1.8	3.9
**4-5**	3.9	1.8	2.4	1.4	2.7	3.1	0.5	2.1	6.3	6.2	3.6	6.4
**4-6**	6.0	5.5	6.6	6.5	5.6	7.5	5.0	6.7	1.9	4.7	3.4	10.2
**4-7**	6.6	3.4	4.2	3.3	5.0	7.5	5.9	7.8	7.1	8.0	3.9	8.6
**5-1**	0.0	0.0	0.0	0.0	0.0	0.0	0.0	0.0	0.0	0.0	0.0	0.0
**5-2**	2.9	0.9	3.3	2.8	1.3	1.1	4.0	4.2	-3.0	-0.9	1.4	3.0
**5-3**	4.2	2.5	7.3	5.9	4.2	3.1	10.5	6.0	-0.5	2.4	4.8	-0.2
**5-4**	6.0	2.8	8.7	5.1	7.1	4.3	9.8	10.3	0.9	-1.4	5.2	7.0
**5-5**	12.6	4.7	16.1	11.9	0.7	10.0	14.9	13.5	0.6	1.1	6.9	7.6
**5-6**	18.1	9.8	13.1	5.6	15.3	10.8	15.8	14.1	16.8	13.1	12.8	13.9
**5-7**	19.5	6.3	11.3	9.7	4.4	11.3	20.3	19.8	19.9	2.5	13.7	12.3
**5-8**	23.9	13.7	16.6	8.0	17.6	17.1	25.5	21.9	17.4	7.3	13.1	14.4
**6-1**	0.0	0.0	0.0	0.0	0.0	0.0	0.0	0.0	0.0	0.0	0.0	0.0
**6-2**	0.7	1.8	5.5	8.1	4.5	2.4	4.1	6.5	6.1	-2.2	-4.3	-2.5
**6-3**	0.8	5.3	8.1	10.7	4.0	3.4	3.8	7.1	8.6	-0.2	-3.4	-3.4
**6-4**	2.6	3.6	2.9	2.9	4.1	2.9	2.9	4.0	1.2	-0.1	4.5	3.2
**6-5**	7.5	5.4	3.2	3.5	11.0	8.3	9.3	14.5	8.5	1.1	1.1	1.6
**6-6**	20.9	13.1	16.1	20.0	12.8	17.0	19.2	22.9	13.4	12.5	15.6	22.7
**6-7**	38.5	36.5	40.4	44.0	35.5	35.7	36.1	40.6	15.0	10.2	21.1	26.3
**7-1**	0.0	0.0	0.0	0.0	0.0	0.0	0.0	0.0		0.0	0.0	0.0
**7-2**	3.7	-2.5	-1.0	1.3	1.6	-0.4	4.1	1.8		6.6	6.3	-1.3
**7-3**	18.5	8.2	8.7	12.5	8.7	7.8	12.7	10.1		13.2	14.7	7.5
**7-4**	11.3	4.9	8.7	12.1	8.7	6.1	11.3	10.1		7.5	11.7	8.5
**7-5**	17.2	5.9	9.2	13.5	7.4	8.1	13.7	11.3		14.4	11.2	8.2
**7-6**	19.3	5.2	12.0	15.7	12.7	8.9	17.0	12.4		11.1	23.1	3.9
**7-7**	22.8	9.5	14.4	17.9	13.6	11.6	20.6	16.4		11.3	22.0	9.3

**7-8**	29.2	16.0	20.2	22.9	15.7	18.9	24.8	24.8		16.3	20.0	16.1
**8-1**	0.0	0.0	0.0	0.0	0.0	0.0	0.0	0.0		0.0	0.0	0.0
**8-2**	0.2	-1.0	2.5	0.7	1.2	1.6	0.9	3.2		7.8	0.0	1.9
**8-3**	2.5	0.9	3.6	2.5	0.4	1.4	-2.7	-2.8		-4.4	0.0	3.0
**8-4**	2.7	4.1	6.6	1.8	4.6	3.1	5.3	4.1		2.6	-2.6	5.2
**8-5**	2.9	3.1	7.4	2.0	3.8	5.6	5.8	6.5		5.6	-1.6	5.2
**8-6**	3.9	4.0	8.8	3.0	2.8	5.2	10.1	8.3		6.2	1.7	5.2
**8-7**	9.4	9.8	11.1	7.4	6.5	9.1	8.4	8.6		9.2	5.5	10.8
**8-8**	12.7	6.8	14.2	3.8	10.2	9.8	7.4	12.7		15.2	9.5	7.3
**9-1**	0.0	0.0	0.0	0.0	0.0	0.0	0.0	0.0	0.0	0.0	0.0	0.0
**9-2**	1.0	-0.4	0.5	1.7	1.8	0.7	0.1	1.2	2.4	0.0	0.0	-2.3
**9-3**	1.9	1.6	1.5	2.6	1.7	1.2	0.9	0.8	0.8	2.1	1.8	-1.5
**9-4**	6.5	3.2	4.1	5.3	4.4	3.4	4.1	4.5	3.2	5.4	5.4	3.5
**10-1**	1.9	0.0	0.0	0.0	0.0	0.0	0.0	0.0		0.0	0.0	0.0
**10-2**	1.7	0.2	0.5	0.1	3.3	3.9	1.6	3.3		2.2	0.9	-1.7
**10-3**	1.3	0.3	0.5	0.2	3.5	3.9	1.5	3.5		3.4	1.2	0.2
**10-4**	2.5	0.5	0.7	0.6	1.8	1.4	0.1	0.8		1.1	1.8	2.8
**10-5**	3.7	0.5	1.1	0.2	4.6	3.0	3.7	3.8		2.0	1.9	-1.0
**10-6**	3.9	1.8	2.5	1.9	4.6	4.7	4.0	7.0		2.5	2.9	1.9
**10-7**	5.1	0.7	1.4	2.5	3.6	4.0	2.9	4.3		0.8	1.6	1.1
**11-1**	0.0	0.0	0.0	0.0	0.0	0.0	0.0	0.0		0.0	0.0	0.0
**11-2**	-0.3	-0.4	0.0	1.2	0.6	-0.5	1.2	-1.1		-0.6	0.6	1.6
**11-3**	0.6	0.9	0.7	0.3	1.6	0.3	1.4	1.2		0.1	0.2	1.8
**11-4**	4.2	0.7	1.0	1.8	0.7	0.5	2.5	1.5		1.5	2.0	2.8
**11-5**	5.8	2.1	2.5	3.5	2.2	2.5	2.3	1.9		2.0	3.3	4.8
**11-6**	6.0	3.4	3.7	4.4	3.3	3.3	3.8	3.2		2.1	3.6	6.8
**12-1**	0.0	0.0	0.0	0.0	0.0	0.0	0.0	0.0	0.0	0.0	0.0	0.0
**12-2**	0.4	1.7	2.3	1.6	0.8	0.0	1.9	3.2	0.8	1.5	1.6	-0.3

**12-3**	1.6	0.2	0.7	1.6	1.6	0.4	0.7	3.5	-0.2	-0.4	0.3	0.1
**12-4**	4.9	3.2	5.4	4.5	4.8	5.3	7.5	8.9	-1.0	1.1	2.9	3.6
**12-5**	7.9	4.8	7.1	6.6	4.4	8.6	10.8	12.6	1.1	3.8	6.2	6.5
**12-6**	7.8	3.3	6.0	7.8	5.6	7.3	10.3	11.4	0.0	3.6	5.1	4.1
**12-7**	8.1	3.2	5.9	5.4	8.3	9.7	11.3	12.4	-0.5	3.7	5.1	4.3
**12-8**	8.6	3.5	6.2	5.7	9.4	7.7	10.6	12.1	0.1	5.3	5.5	4.8
**13-1**	0.0	0.0	0.0	0.0	0.0	0.0	0.0	0.0	0.0	0.0	0.0	0.0
**13-2**	0.9	0.1	1.0	1.2	0.9	1.2	0.9	0.8	0.9	1.1	0.9	0.8
**13-3**	1.4	2.0	1.3	0.7	-1.6	0.6	-1.5	-0.8	3.1	2.6	1.2	-0.7
**13-4**	1.2	0.7	1.1	1.5	2.4	2.5	2.6	2.1	2.3	2.2	1.3	0.8
**13-5**	0.7	3.2	2.4	1.8	-0.4	1.5	-0.6	0.7	0.0	1.0	1.5	-1.0
**13-6**	1.1	3.4	2.5	2.2	-0.2	1.8	-0.3	0.7	0.3	2.1	1.5	-0.3
**13-7**	1.5	6.7	6.7	5.8	1.8	4.8	-0.6	0.4	2.0	2.8	2.4	-1.7
**13-8**	3.1	2.1	0.1	0.4	-0.3	1.7	-1.4	-1.4	3.6	2.9	2.0	6.3
**14-1**	0.0	0.0	0.0	0.0	0.0	0.0	0.0	0.0	0.0	0.0	0.0	0.0
**14-2**	-0.8	-3.2	-2.3	0.7	-1.0	-1.0	0.8	-2.5	-2.8	2.1	-0.3	-5.9
**14-3**	2.0	1.7	2.6	-1.8	-0.8	1.3	5.1	1.2	0.7	4.7	2.8	-2.8
**14-4**	3.0	1.3	2.7	2.4	0.6	3.8	4.7	1.4	0.4	4.3	3.5	-2.9
**14-5**	3.0	0.1	2.0	-2.9	-2.6	3.7	2.3	0.8	0.7	4.3	2.9	-2.3
**14-6**	3.1	-2.0	0.1	-3.1	-3.8	-0.4	5.2	5.4	0.4	5.9	3.9	0.8
**14-7**	6.2	3.5	2.5	3.3	4.4	6.4	8.3	4.1	1.8	5.4	6.1	2.9
**14-8**	6.0	3.8	-0.5	-0.1	3.0	3.9	-0.6	-6.1	3.1	8.6	4.6	7.1
**15-1**	0.0	0.0	0.0	0.0	0.0	0.0	0.0	0.0	0.0	0.0	0.0	0.0
**15-2**	1.2	0.6	0.4	0.5	0.3	0.3	0.4	0.6	2.3	2.1	0.2	-0.9
**15-3**	3.1	0.4	3.4	6.9	4.7	3.4	9.4	2.8	0.6	-1.3	2.8	0.0
**15-4**	5.4	-0.1	4.0	6.1	1.6	-0.3	1.4	3.8	1.0	2.3	2.3	-1.2
**15-5**	9.9	3.9	14.0	16.4	9.5	7.1	13.0	15.6	2.6	1.9	6.1	5.0
**15-6**	14.5	7.9	19.1	21.2	15.1	6.6	18.0	20.6	7.4	8.9	11.7	10.0

**15-7**	17.9	1.1	20.6	15.2	21.5	15.8	21.4	23.9	5.1	7.2	13.0	7.7
**15-8**	17.4	14.7	17.3	23.0	16.4	17.7	13.8	17.7	9.0	10.3	5.2	15.0
**16-1**	0.0	0.0	0.0	0.0	0.0	0.0	0.0	0.0		0.0	0.0	0.0
**16-2**	0.0	0.0	0.0	0.0	0.3	0.0	-0.9	0.0		0.0	0.0	0.0
**16-3**	0.2	1.1	1.0	1.0	0.6	1.7	-2.1	-1.3		-2.2	1.1	-0.4
**16-4**	0.2	1.1	1.0	1.0	0.7	1.6	-2.2	-1.4		-2.2	1.1	-0.4
**17-1**	0.0	0.0	0.0	0.0	0.0	0.0	0.0	0.0	0.0	0.0	0.0	0.0
**17-2**	0.7	0.1	0.1	-0.8	-0.4	1.9	0.1	0.3	0.9	0.7	0.7	1.8
**17-3**	2.4	2.2	3.7	3.6	2.7	2.3	3.2	6.5	1.4	2.0	1.1	3.0
**17-4**	4.2	0.3	2.5	3.2	2.8	3.0	3.9	2.9	4.1	2.5	4.0	-0.8
**17-5**	3.7	1.3	3.1	2.7	2.2	4.0	3.8	3.2	4.4	2.4	3.6	0.9
**17-6**	4.9	2.2	2.9	2.8	2.4	4.0	4.3	4.2	4.1	3.0	2.6	5.0
**17-7**	9.4	3.2	4.7	4.8	6.6	6.8	4.9	9.1	10.7	5.2	9.2	6.2
**17-8**	9.6	2.2	5.8	5.7	5.7	7.8	11.6	9.0	12.7	10.2	10.9	5.1
**18-1**	0.0	0.0	0.0	0.0	0.0	0.0	0.0	0.0		0.0	0.0	0.0
**18-2**	0.1	0.0	0.0	0.0	0.0	0.0	-0.2	0.0		0.0	0.0	2.0
**19-1**	0.0	0.0	0.0	0.0	0.0	0.0	0.0	0.0		0.0	0.0	0.0
**19-2**	3.1	8.2	8.3	9.8	6.2	8.0	0.6	2.4		3.0	4.5	8.4
**19-3**	3.7	3.9	7.4	7.9	4.3	5.6	6.4	9.3		-1.2	1.6	2.9
**19-4**	3.6	4.0	7.4	7.9	4.2	5.5	5.8	8.9		-1.1	1.6	2.5
**19-5**	3.9	4.2	7.6	8.1	4.3	5.9	5.9	9.1		-0.9	1.5	3.2
**19-6**	3.8	4.1	7.6	7.9	4.2	5.8	6.2	9.4		-1.5	1.5	2.1
**19-7**	8.2	10.2	12.0	12.9	8.4	11.0	3.7	8.7		2.2	4.0	10.6
**19-8**	9.6	10.2	13.0	14.0	10.1	12.7	9.0	12.2		2.7	4.3	10.0
**20-1**	0.0	0.0	0.0	0.0	0.0	0.0	0.0	0.0		0.0	0.0	0.0
**20-2**	0.1	0.3	0.3	0.4	0.3	0.2	0.4	0.6		0.0	-0.1	-0.8
**20-3**	0.0	0.4	0.3	0.4	0.1	0.3	0.0	-1.1		-0.1	-0.2	-0.1
**20-4**	0.2	0.7	0.6	0.7	0.5	0.2	-0.8	-0.4		0.1	0.0	0.4

**20-5**	3.4	3.2	3.9	3.4	4.9	5.5	3.0	5.8	2.6	3.7	3.1
**20-6**	4.1	4.3	4.5	3.9	5.4	6.4	4.1	6.9	3.1	3.6	3.4
**20-7**	10.1	10.1	11.3	11.5	11.2	12.6	9.3	10.1	2.3	5.1	-1.8
**20-8**	31.0	19.9	31.6	30.1	33.4	36.9	29.0	24.9	8.1	6.4	11.5
